# Development and validation of a meal quality index with applications to NHANES 2005-2014

**DOI:** 10.1371/journal.pone.0244391

**Published:** 2020-12-22

**Authors:** Fabio Mainardi, Daniela Prozorovscaia, Gary Sweeney, Hilary Green

**Affiliations:** 1 Nestlé Research, Lausanne, Switzerland; 2 British Dietetics Association, Birmingham, United Kingdom; The University of Hong Kong, HONG KONG

## Abstract

The Meal Balance Index (MBI) assesses the nutritional quality and balance of meals. It is a score between 0 and 100 that takes into account both shortfall and excess nutrients, adjusted for the energy content of the meal. In the present study the score was applied to 147849 meals reported in the National Health and Nutrition Examination Surveys (NHANES) 2005-2014 in order to evaluate its validity and compare against exemplary meals designed as part of 24h diets that meet US dietary guidelines. Meals from exemplary menu plans developed by nutrition experts scored on average 76±14 (mean ± standard deviation) whereas those of NHANES participants scored 45±14. Scores of breakfast, lunch, dinner, snack, considered jointly as independent variables, were moderately but positively and significantly associated with the Healthy Eating Index (Pearson correlation 0.6). MBI scores were significantly associated with the density of positive micronutrients (e.g. Vit A, Vit C) and favorable food groups (e.g. fruits, whole grains) not directly included in the MBI algorithm. The MBI is a valid tool to assess the nutritional quality of meals reported in the US population and if applied to culinary recipe websites could potentially help users to understand which meals are nutritionally balanced. Choice of more balanced individual meals can guide healthier cooking and eating.

## Introduction

In 2017, non-communicable diseases (NCDs) accounted for 73.4% of total deaths worldwide [1]. Since dietary behaviors are considered one of the main risk factors for NCDs [2] various initiatives have been introduced by public health authorities to promote healthy eating [3, 4]. For example, most countries have developed food-based dietary guidelines (FBDG) to help the general population improve dietary behaviors and food choices [5, 6].

Moreover, there is a growing body of evidence showing that meal patterns influence dietary intake both quantitatively and qualitatively [7, 8] and that understanding meal patterns can bring valuable insights to dietary advice, assisting individuals in meeting recommended daily intakes of foods and nutrients [9]. Quoting from the Dietary Guidelines for Americans, “eating patterns are the result of choices on multiple eating occasions over time, both at home and away from home. As a result, individuals have many opportunities to make shifts to improve eating patterns” [10].

Despite this evidence, few studies to date have investigated meal quality indicators [11]. Meal quality indicators described in the literature were often developed to assess the nutritional quality of specific meals (e.g. breakfast) and generally lack a comprehensive methodology for internal validation [11]. To our knowledge, all meal quality indicators previously developed were hybrid tools—meaning that the algorithms were based on both nutrients and food groups [11]. To apply hybrid algorithms to dietary intake data or to recipe data, conversion of foods, mixed dishes, food products or ingredients to food group data is required. This can be done through the Food Patterns Equivalents Database (FPED) in the United States (US), which converts mixed dishes and processed foods into food groups, however these tools are not available for most other countries.

While dietary reference intakes and other dietary recommendations are expressed as daily recommendations, there are no generally accepted recommendations for individual eating occasions; however, dietary advice might be more practical and easier to follow if given for meals and snacks. Public health authorities advocate cooking more meals at home, and one popular and modern way of seeking recipe inspiration is through online culinary recipe websites. Recent research shows that the nutritional quality of online “main dish” recipes might have worse nutritional profiles than ready-to-eat meals from supermarkets [12]. Thus, it is of public health interest that consumers have easy ways to assess and understand whether their meals, and their preferred online culinary recipes are nutritionally balanced.

Our starting point was a previously published diet quality index, based on 16 shortfall and overconsumed nutrients reported in the 2015 US Dietary Guidelines [13]. The Meal Balance Index (MBI) is an adaptation of that scoring system using only 9 nutrients and rates the nutritional quality of meals instead of 24h diets. MBI can be applicable either as a research tool on population data (as in the present study), or as a consumer-facing tool to aid in choosing healthy meals (e.g. online recipes, canteen menus).

## Materials and methods

### Description of the algorithm

The MBI is an adaptation of the 24h diet score previously developed and validated by Mainardi et al [14]. Nutrients that were selected for inclusion in the MBI are 9 out of the 16 nutrients used in that model. These 9 nutrients are: protein, total fat, fiber, potassium, calcium, iron, sodium, added sugars and saturated fat.

The methodology used to select those 9 nutrients from the initial list of 16 was partly data-driven and partly motivated by prior assumption. Saturated fat, sodium and added sugars were kept in the scoring system, as they are recognized as nutrients of concern for public health [13]. Vitamin D was removed due to lack of feasibility of application, because it is very often unavailable in food databases.

**Fig 1:**
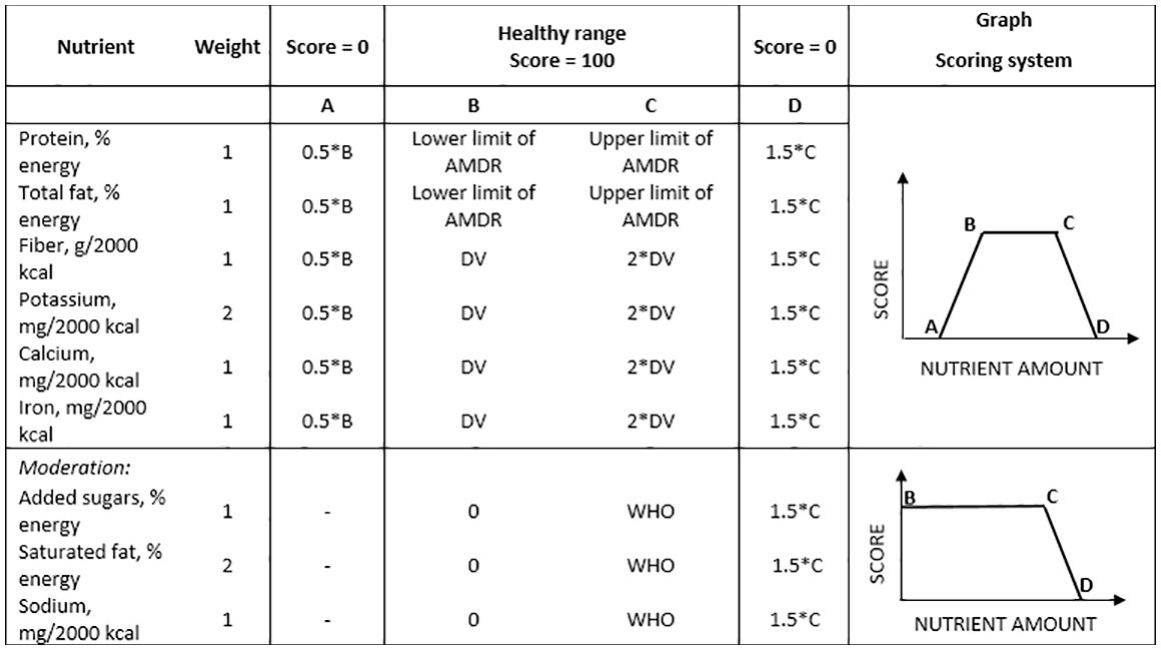
MBI scoring system (DV: Daily value, AMDR: Acceptable macronutrient distribution range). The charts on the right illustrate the way that points are awarded for protein, total fat, fiber, potassium, calcium, iron (top), and for sodium, added sugars and saturated fat (bottom). Healthy ranges correspond to the interval [B, C].

The choice of the remaining nutrients was data-driven, according to the following process. Like the Healthy Eating Index, the MBI is calculated as a weighted average of several sub-scores. Therefore, the algorithm is determined by the choice of the nutrients, how they are scored, and the choice of their respective weights in the average. The calculation of the individual sub-scores is detailed below and summarized in Fig 1. In order to determine an optimal choice of the weights, we ran systematically the scoring algorithm on NHANES meals, choosing for each of the candidate nutrients, one of three possible weights: 0, 1, 2. A zero weight means that the corresponding nutrient is excluded from the score, so that this process actually selects the nutrients to be used in the algorithm. There were 177147 possible choices for the 9 weights, and we used as criteria for selecting them the following constraints:

- scores should be (approximately) symmetrically distributed;
- odds-ratios for predicting intake of the following: whole fruit, whole grain, ratio of oils to solid fats, red and orange vegetables, processed meat, dark-green vegetables, should be statistically significant (5%);
- Spearman correlation with the 16-nutrients score should be above 0.8.

Out of the possible combinations of weights satisfying all these constraints, we selected the one that is most closely aligned with the new US food label regulations [15].

A potential concern is the lack of vitamins in the score; for this reason, we report in the results section the association between the score and some vitamins, and the correlation between the score based on 9 nutrients and the score based on 16 nutrients.

The Meal Balance Index uses the same nutrient healthy ranges principle as in the model for 24h diets previously developed by Mainardi et al [14]. The scoring system is summarized in Fig 1 and is adapted from the same reference [14]. The healthy ranges for fiber, potassium, calcium and iron are based on daily values (DVs). We define the healthy range as 100–200% DV. For sodium, saturated fat and added sugars the healthy range was defined as 0–100% of levels recommended by the World Health Organization. The healthy range for macronutrients was defined using the Acceptable Macronutrient Distribution Ranges (AMDRs) recommended by the Food and Nutrition Board, Institute of Medicine, National Academies. For insufficient micronutrient intakes, a score of zero was given when intake was ≤(0.5 × DV). For micronutrient intakes above the healthy range, a score of zero was given when intake was ≥(1.5 × the upper healthy range). The present upper limit (200% DRI) is distinct from, and lower than, the Tolerable Upper Limit (TUL) established for some nutrients by regulatory authorities and expert panels.

To obtain the score of each nutrient, first, its amount is scaled to 2000 kcals, in order to express the score in terms of nutrient density. Then, the scaled amount of each nutrient in the model is compared to the amount as defined by the healthy range expected in 2000 kcals. Each nutrient is scored independently and the MBI is their weighted average.

### Validation methodology

In Table 1 we summarize the steps taken to assess the validity of the MBI.

**Table 1:** Strategies used to validate the Meal Balance Index (MBI).

Question	Strategy
Does the score reflect the fact that the average US adult does not meet the dietary guidelines?	Compute scores of meals from exemplary menu plans designed according to dietary guidelines and compare with scores of meals actually consumed in the US population.
Does the score reflect the overall diet quality?	Compare with the Healthy Eating Index
Does the scoring system correlate with the content of nutrients and food groups not directly included in the calculation?	Compare the densities of food groups and nutrients across tertiles of meal scores

First, we scored meals from exemplary menu plans, designed by nutrition experts, using a step by step methodology aligned with the dietary guidelines key requirements and limitations (Table 2). We included a 2-weeks menu plan for each of the following three eating patterns: the healthy US, Vegetarian and Mediterranean, proposed as examples in the Dietary Guidelines for Americans [10]. In addition, we included a 1-week menu plan developed according to the 2015 Mexican dietary guidelines [16]. Meals scores of all menu plans were compared to the scores of meals as reported by 2005-2014 NHANES participants (4 years and older). Energy intakes from NHANES meals have very right-skewed distributions and outliers may potentially affect the analysis. A pragmatic, and realistic, range of 200-1200 kcal was used to define which NHANES meals would be included in the analysis. This range was chosen after looking at the distributions, and after checking that slightly different choices would not affect the main conclusion of the analysis.

**Table 2:** Key criteria used to generate the exemplary menu plans.

Factor	2015-2020 Dietary Guidelines for Americans	2015 Mexico Dietary Guidelines
Energy	1800-2200 kcals	2000 kcals
Sodium	≤ 2300 mg	1600 mg
Saturated fat	≤ 10% of daily Kcals	≤ 7% of daily Kcals
Added sugars	≤ 10% of daily Kcals	≤ 10% of daily Kcals
Dairy	Low or fat-free choices	Low or fat-free choices
Meat and poultry	Lean cut choices (e.g. ≥ 95% lean ground beef)	3.5 portions per day (half of the amount consumed per day should come from lean cut choices)
Oils	≤ 27 g	≤ 4 portions
Daily calories for other uses	≤ 270 Kcals (US); ≤ 290 Kcals (Vegetarian); ≤ 260 Kcals (Mediterranean)	No details

Second, we considered daily food intakes from NHANES 2011-2012, day 1 of recall, and scored them with the Healthy Eating Index (HEI) 2010. Since the definition of the Healthy Eating Index has changed over time, we decided to limit this analysis to the 2010 version. We hypothesized that the combination of the MBI scores of meals consumed in a day should be positively associated with the Healthy Eating Index of the corresponding daily intakes. To test this hypothesis, we evaluated a linear regression model, with HEI as dependent variable and the MBI scores of the main eating occasions as independent variables, after adjusting for daily energy intake, age and gender. We included age and gender as covariates because they were shown to be associated with the Healthy Eating Index [17], so they could be potential confounders.

A randomly selected 70% of observations was used to fit the linear regression model, after checking that this selection was unbiased with respect to the variables of interest. The remaining 30% of data was used to validate the model.

The coefficients for the meal scores in the regression model are expected to be all positive and can be interpreted as the expected increase in HEI score per unit increase of the meal score, if all other co-variates are kept constant. To assess the predictive value of the model, we applied the fitted linear equation to the remaining 30% of the data (which was not used to fit the linear regression model) and compared the resulting predicted values with the actual HEI. In order to mitigate the effect of multi-collinearity, we introduced a Ridge regularization term in the regression model [18], with parameter selected by cross-validation. *p*-values for the coefficients in Ridge regressions were calculated according to Cule et al [19].

Finally, we tested if MBI meal scores are associated with the content of nutrients and specific foods in the meals of NHANES participants 4 years and older. Magnesium, vitamin A, vitamin C and vitamin E are known to be commonly under-consumed in the U.S., but they are not included in the calculation of the score. Therefore, we tested the association between the content of these nutrients and the score.

### Data and software

Menu plans were designed according to four dietary patterns: healthy US-style, Mediterranean, Vegetarian, Mexican. The first three were developed following tables A3-1, A4-1, A5-1 in the dietary guidelines for Americans 2015-2020, 2000 kcals, the fourth was based on Mexican guidelines [10, 16]. The Healthy U.S.-Style Pattern is based on the types and proportions of foods Americans typically consume, but in nutrient-dense forms and appropriate amounts. The Healthy Vegetarian Pattern is adapted from the Healthy U.S.-Style Pattern, modifying amounts recommended from some food groups to more closely reflect eating patterns reported by self-identified vegetarians in the National Health and Nutrition Examination Survey (NHANES). The Healthy Mediterranean-Style Pattern contains more fruits and seafood and less dairy than does the Healthy U.S.-Style Pattern. The menu plans are available in the S1 File.

Nutrient content was based on USDA Standard Reference Database, version 28. Dietary intake data were obtained from the NHANES in-person 24 h recalls (2 days of recall); all NHANES data are publicly available on the NCHS and USDA websites. We used the cycles from 2005 to 2014, subjects of age at least 4 y. This information included names of foods, times they were consumed and name of eating occasion.

The Food Patterns Equivalents Database (FPED [20]) was used to evaluate consumption in terms of food group equivalent servings (cup-equivalents for fruits and vegetables, oz-equivalents for protein foods and grains). Data for added sugars were also extracted from FPED. For added sugars, we used the conversion factor 1 teaspoon = 4.2 grams. Macronutrients were converted from grams to kcals using the standard Atwater conversion factors (4 for carbohydrate and protein, 9 for fat).

All the data analysis was performed with the software R, version 3.5.0.

## Results

### Reduction from 16 to 9 nutrients

**Fig 2:**
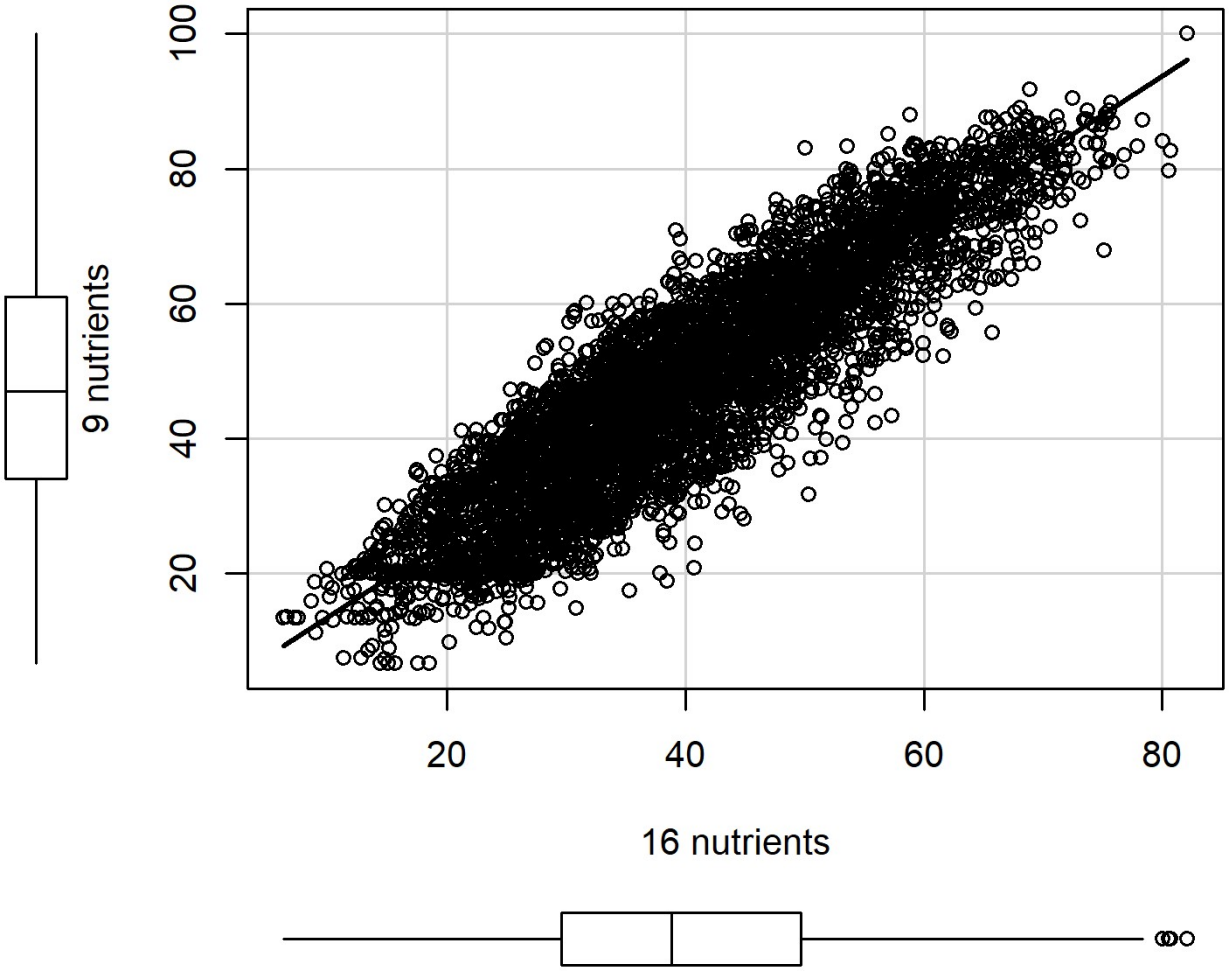
Comparison of lunch scores, using 16 nutrients (horizontal axis) and 9 nutrients. NHANES data. Spearman correlation = 0.89.

Fig 2 compares the scores of lunch meals from NHANES, age 4+, calculated as the unweighted average of 16 nutrient scores [14] (horizontal axis), and the weighted average of the selected 9 nutrient scores, as defined in Fig 1. The average and standard deviation on the x-axis and y-axis were 40 ± 14 and 48 ± 18, respectively.

Similar results, not shown here, were found for the other main eating occasions: breakfast and dinner.

### MBI scores of NHANES meals and exemplary menu plans

**Fig 3:**
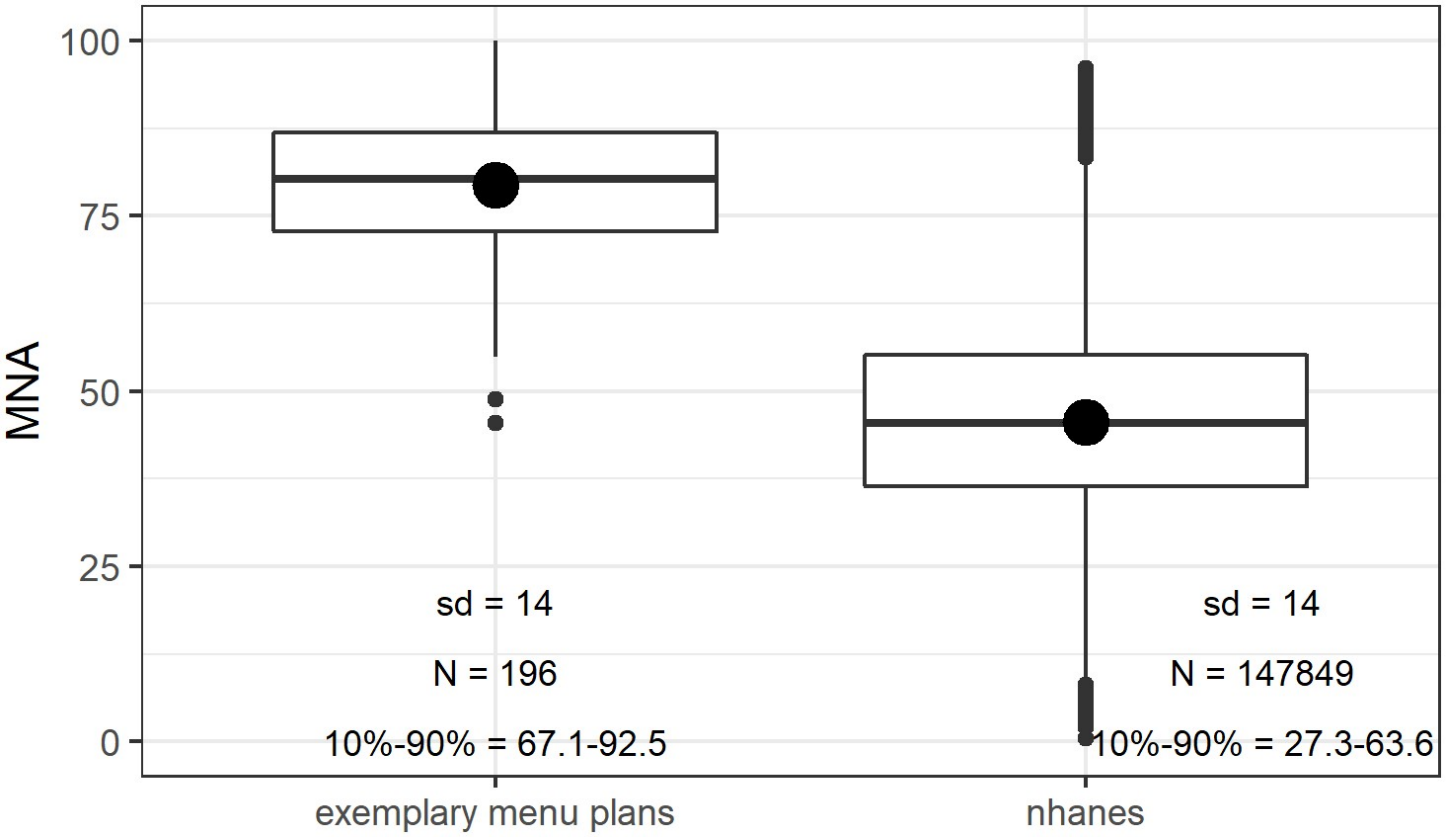
Comparison of meal scores from exemplary menu plans (left) and from NHANES meals (breakfast, lunch, snack, dinner, age 4+). Standard deviations, sample sizes and 10%-90% percentiles ranges are shown.

Meals with energy between 200 and 1200 kcals, consumed by males and females at least 4 years old scored, on average (SD) 45.5 (14). Meals from exemplary menu plans scored on average (SD) 75.9 (14). Their respective distributions are shown in Fig 3 as boxplots.

In Table 3 we summarize the scores of the main eating occasions in NHANES and in the menu plans. Sample sizes are: N = 14 for each meal in the menu plans, except for the Mexican (N = 7). From NHANES, we had the following sample sizes: N = 39630 (breakfast), N = 35687 (lunch), N = 38976 (snack), N = 33556 (dinner).

**Table 3:** Average meal scores and standard deviations from exemplary menu plans and NHANES.

Meal	Mexican	Vegetarian	Med.	US-style	NHANES
Breakfast	76 (11)	89 (8)	75 (17)	85 (7)	49 (14)
Lunch	74 (10)	79 (11)	76 (11)	79 (10)	46 (13)
Snack	78 (7)	77 (7)	75 (9)	67 (11)	40 (14)
Dinner	74 (4)	83 (5)	79 (9)	83 (10)	46 (13)

### Measuring association of meal scores with diet quality

Estimates for the coefficients of the linear regression model, with standard errors and significance, are reported in Table 4. All the variables, except gender, were statistically significant (*p* < 0.05).

**Table 4:** Linear regression model for HEI2010 as a function of MBI.

	Coefficient estimate	Standard error	p-value
Breakfast	0.27	0.04	3.53e-11
Lunch	0.20	0.04	4.71e-06
Snack	0.31	0.04	4.22e-15
Dinner	0.09	0.04	0.0441
Age	0.16	0.03	2.73e-10
Gender (female)	-0.14	1.15	0.9009
Energy	-0.002	0.001	0.0155
Observations	2072		

The optimal Ridge regularization parameter was selected by cross-validation and was estimated to be 0.036.

**Fig 4:**
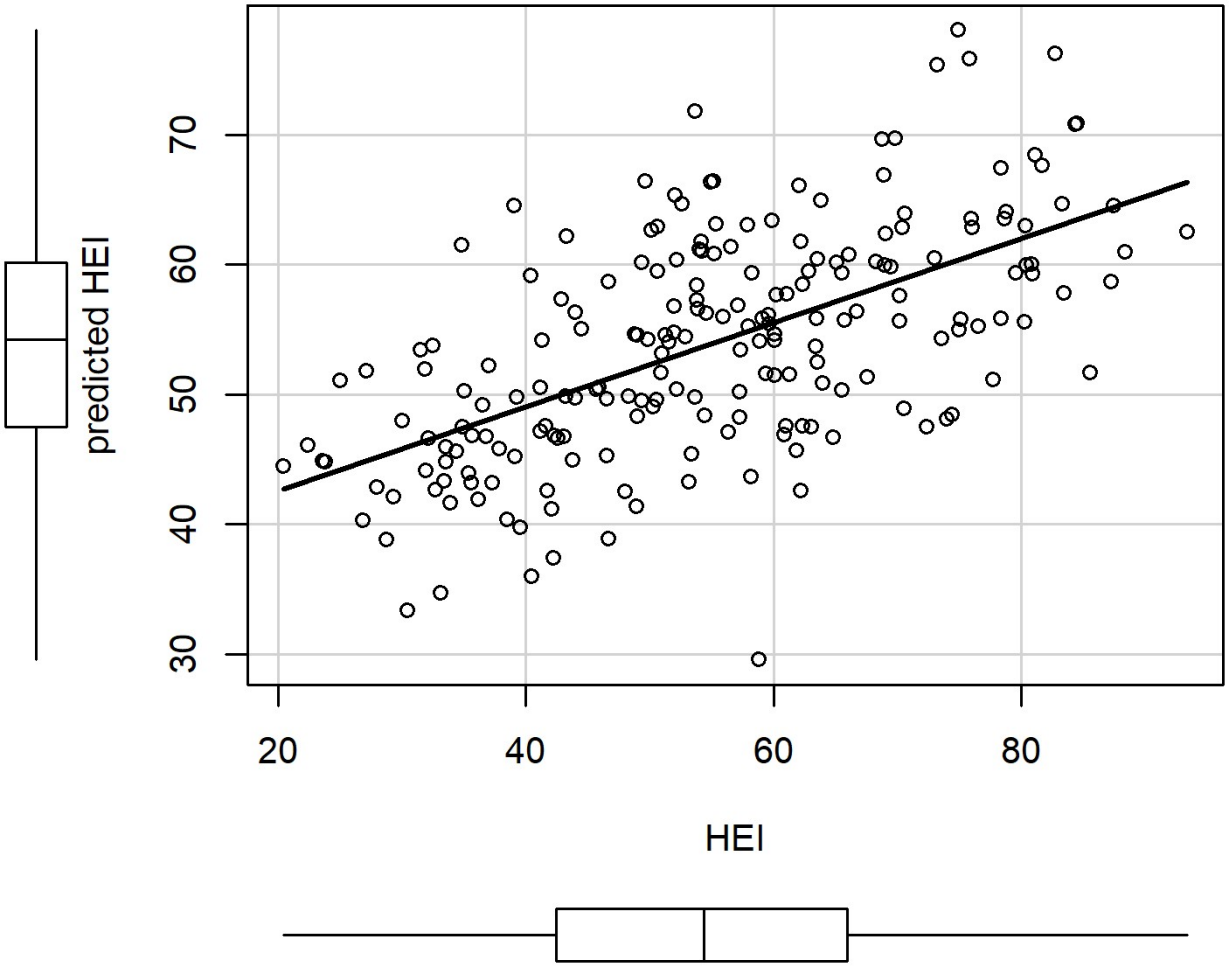
Scatterplot of HEI2010 against prediction from the linear model, using 30% of the data as a test set (204 observations). Pearson correlation = 0.6.

When we applied the linear equation to the remaining 30% of the data, and compared the result with the HEI2010 (Fig 4), we observed that the Pearson correlation between the linear regression model and the actual HEI was 0.6.

### Association with food groups and nutrient density

As explained in the methods, we report the association between meal scores and the content of food groups and nutrients. Table 5 describes the food group profiles of the main eating occasions in NHANES.

**Table 5:** Average content and standard deviation of food groups in the main eating occasions and NHANES.

	Breakfast (N = 39630)	Dinner (N = 35687)	Lunch (N = 38976)	Snack (N = 33556)
Energy (kcal)	445 (208)	561 (257)	512 (236)	512 (247)
Dairy (cup-eq)	0.7 (0.8)	0.4 (0.7)	0.5 (0.7)	0.4 (0.6)
Grains (oz-eq)	1.9 (1.5)	2.1 (1.8)	2.0 (1.6)	1.5 (1.5)
Whole grains (oz-eq)	0.6 (1.0)	0.2 (0.6)	0.2 (0.6)	0.2 (0.6)
Fruit (cup-eq)	0.4 (0.7)	0.2 (0.5)	0.3 (0.6)	0.5 (0.9)
Vegetables (cup-eq)	0.1 (0.3)	0.9 (1.2)	0.5 (0.8)	0.1 (0.4)
Meat and seafood (oz-eq)	0.09 (0.5)	0.6 (1.4)	0.5 (1.1)	0.07 (0.4)

In Table 6 scores and amounts refer to breakfasts from NHANES 2005-2015, day 1 of recall, adjusted for energy.

**Table 6:** Association between breakfast scores and density of food groups and nutrients.

Food groups	Tertile of meal score			Unit	Recommendation.
	[0, 43]	(43, 55]	(55, 100]		
Dairy	2.8^*a*^	3.5^*b*^	3.4^*b*^	cup-eq/2000 kcal	3 cup-eq
Fruits	1^a^	2^b^	2.6^*c*^	cup-eq/2000 kcal	2 cup-eq
Citrus, melons, berries	0.1^*a*^	0.2^*b*^	0.4^*c*^	cup-eq/2000 kcal	
Grains	7.6^*a*^	8.8^*b*^	9.6^*c*^	oz-eq/2000 kcal	6 oz-eq
Whole Grains	1.0^*a*^	2.7^*b*^	4.2^*c*^	oz-eq/2000 kcal	3 oz-eq
Nuts and seeds	0.2^*a*^	0.4^*b*^	0.6^*c*^	oz-eq/2000 kcal	
Solid fats	47^a^	26^b^	20^c^	g/2000 kcal	
**Vitamins**					
Folate	565^a^	890^b^	854^c^	*μ*g/2000 kcal	400 *μ*g
Vit A	893^a^	1315^b^	1314^c^	*μ*g/2000 kcal	900 *μ*g
Vit C	89^a^	151^b^	161^c^	mg/2000 kcal	90 mg
Vit E	6.2^*a*^	7.4^*a*^	7.7^*b*^	*μ*g /2000 kcal	15 *μ*g
Vit K	47^a^	42^b^	40^c^	*μ*g/2000 kcal	120 *μ*g
**Minerals**					
Copper	0.9^*a*^	1.2^*b*^	1.4^*c*^	mg/2000 kcal	0.9 mg
Magnesium	253^a^	362^b^	417^c^	mg/2000 kcal	420 mg
Zinc	7^a^	10^b^	12^c^	mg/2000 kcal	11 mg
**Macronutrients**					
Energy	446^a^	436^b^	454^c^	kcal	
Total sugar	156^a^	164^b^	158^c^	g/2000 kcal	
Cholesterol	188^a^	119^b^	96^c^	mg/2000 kcal	

All units are expressed per 2000 kcal. Different letters indicate statistically different distributions (pairwise Wilcoxon test, 95% confidence level). Recommendations based on the US dietary guidelines, when applicable.

Similarly, Table 7 illustrates the results for lunch. Similar results, not shown here, hold for dinner and snack.

**Table 7:** Association between lunch scores and density of food groups and nutrients.

Food groups	Tertile of meal score			Unit	Recommendation
	[0, 40]	(40, 52]	(52, 100]		
Dairy	2.3^*a*^	2^b^	1.9^*b*^	cup-eq/2000 kcal	3 cup-eq
Fruits	0.6^*a*^	1^b^	1.9^*c*^	cup-eq/2000 kcal	2 cup-eq
Citrus, melons, berries	0.1^*a*^	0.2^*b*^	0.4^*c*^	cup-eq/2000 kcal	
Vegetables	1.8^*a*^	2.2^*b*^	2.4^*c*^	cup-eq/2000 kcal	2.5 cup-eq
Legumes	0.05^*a*^	0.13^*b*^	0.18^*c*^	cup-eq/2000 kcal	
Grains	7^a^	8^b^	10^c^	oz-eq/2000 kcal	6 oz-eq
Whole Grains	0.5^*a*^	0.9^*b*^	1.6^*c*^	oz-eq/2000 kcal	3 oz-eq
Nuts and seeds	0.2^*a*^	0.5^*b*^	0.8^*c*^	oz-eq/2000 kcal	
Solid fats	40^a^	27^b^	19^c^	g/2000 kcal	
**Vitamins**					
Folate	323^a^	428^b^	490^c^	*μ*g/2000 kcal	400 *μ*g
Vit A	572^a^	611^b^	690^c^	*μ*g/2000 kcal	900 *μ*g
Vit C	70^a^	95^b^	125^c^	mg/2000 kcal	90 mg
Vit E	7^a^	7.5^*b*^	8.6^*c*^	*μ*g /2000 kcal	15 *μ*g
Vit K	132^a^	145^b^	161^c^	*μ*g/2000 kcal	120 *μ*g
**Minerals**					
Copper	1.0^*a*^	1.3^*b*^	1.5^*c*^	mg/2000 kcal	0.9 mg
Magnesium	211^a^	272^b^	330^c^	mg/2000 kcal	420 mg
Zinc	8^a^	9^b^	10^c^	mg/2000 kcal	11 mg
**Macronutrients**					
Energy	519^a^	521^b^	496^c^	kcal	
Total sugar	132^a^	118^b^	108^c^	g/2000 kcal	
Cholesterol	151^a^	122^b^	106^c^	mg/2000 kcal	

All units are expressed per 2000 kcals. Different letters indicate statistically different distributions (pairwise Wilcoxon test, 95% confidence level). Recommendations based on the US dietary guidelines, when applicable.

## Discussion

### Reduction from 16 to 9 nutrients

The original 16 nutrients included more macronutrients, more minerals and vitamins and the rationale for their selection is detailed elsewhere [14]. The main reason for reducing the selection of nutrients (from 16 to 9) was the low coverage of some micronutrients in many food composition databases, therefore limiting applicability and usability. For example, in the USDA standard reference database, the proportion of missing values is 40% for vitamin K, 37% for vitamin D, 33% for vitamin E, 17% for vitamin A, 9% for vitamin C, 8% for magnesium. For comparison, calcium and iron are missing only 4% and 1.6% of the values, respectively.

Added sugars are not always available in food databases, while critical for public health, thus were kept in the reduced scoring tool. However, added sugars intakes are available for NHANES data and the introduction of newly-revised labelling information in the US now includes added sugars as mandatory. At the same time, databases are increasingly reporting added sugars and several systematic methodologies have been published to impute added sugars in food composition databases [21]. Saturated fats and sodium are generally well represented in both food databases and product labels. The 9 selected nutrients were actually made mandatory in the updated FDA regulations for food labeling in the United States [13].

Fig 2 compares the score, based on 9 nutrients, with the score based on 16 nutrients. The Spearman correlation is 0.89 and is statistically significant. In addition, the score with 9 nutrients spans a wider numerical range, with average shifted by 8 points, so that the 9-nutrients score is approximately a re-scaled version of the 16-nutrients score. In particular, rankings of meals according to the two versions of the score will be concordant.

### Association with diet quality

It is known from previous studies that less than 20% of U.S. adults meet the U.S. Department of Agriculture dietary guidelines [22]. Correspondingly, meals from exemplary menu plans achieved significantly higher scores than the average NHANES meals (Table 4 and Fig 3). The menu plans used in this analysis were designed independently of the development of the MBI, so this comparison supports the hypothesis that meals of high nutritional quality, as measured by MBI, are a signature of a nutritionally adequate diet.

Lower nutritional quality of NHANES meals (as assessed by the MBI score) was associated with lower overall diet quality (as assessed by the HEI). Table 4 and Fig 4 show how the MBI is associated with the HEI. Scores of breakfast, lunch, dinner, snack, considered jointly as independent variables, were moderately but positively associated with HEI. For example, according to Table 4, an increase of 5 points in the scores of each eating occasion, would increase on average the HEI by 4 points, based on the linear regression model (for fixed values of energy, age and gender).

The HEI calculation is based on the total intakes of food groups and nutrients (sodium, saturated fat and added sugars) in a day, and it does not take into account how these intakes are distributed among the different eating occasions. Therefore, a given 24h diet can correspond to many possible patterns of meals. This variability might explain the modest correlation observed in Fig 4. Similar correlations were also observed between diet quality scores and meal quality scores in a previous study by K. Murakami [23], where a meal quality index based on the FSA score was used as independent variable in a linear model with the Mediterranean Diet Score as response variable.

Similar to the findings reported by Murakami [23], the coefficients in Table 4 suggest that the strength of the associations with overall diet quality is different between main eating occasions and snacks. In addition, our results suggest significant differences between breakfast, lunch and dinner. To our knowledge, this has not been investigated before.

### MBI correlates with the content in meals of food groups and nutrients of public health concern

Potassium, dietary fiber, magnesium, calcium, and vitamins A, C, D, and E were identified as under-consumed in the US [13]. Among these, only potassium, fiber and calcium are included in the calculation of the MBI. Nonetheless, meals with higher scores have higher density of most of the minerals and vitamins we looked at (Tables 6 and 7). On the other hand, for vitamin K we observed a negative association with breakfast scores, but a positive association with lunch scores, probably reflecting different food choices in the two eating occasions [24]. In fact, the main food source of vitamin K is dark-green vegetables, and from Table 5 it appears that the daily intakes of vegetables come primarily from lunch and dinner. Along the same lines, the density of total sugar was positively associated with breakfast scores, but negatively associated with lunch scores, perhaps a consequence of higher intakes of dairy and fruits associated with breakfast [24].

We also observed an increasing trend for several food groups, in particular fruits, dairy and whole grains. In particular, even if the MBI does not include a component for fruits and vegetables, or for vitamins, Tables 6 and 7 indicate that following nutritional advice based on the MBI, will generally lead to an increase in the intake of fruits and vitamins, as well as of magnesium.

### Strengths and limitations

The strengths of this study include a comprehensive validation, to assess the association of meal quality with diet quality, following an approach combining ‘ideal’ menu plans, designed in compliance with dietary guidelines, and population data, reflecting eating patterns of the US adult population. However, there are some limitations. The cross-sectional nature of the data precluded the possibility of detecting associations between meal patterns and health outcomes or body measures, like glycaemia or waist circumference. Another limitation to mention is that meal patterns might be influenced by socio-economic factors, like the education level, income, degree of acculturation or ethnicity. We did not investigate these aspects in the present study. Moreover, different meal patterns (e.g. breakfast skipping) were not considered in our analysis. In the future it would also be interesting to correlate the MBI with other diet quality indicators (eg DASH score, NRF).

## Conclusions

The present study has shown that MBI is a valid measure of the nutritional quality of meals, and proposed a novel approach of looking at meal patterns as an indicator of diet quality. As such, the MBI can be applied either as a research tool in population studies, or for individual education and guidance for cooking and eating. Many nutrient profiling models focus on rating the nutritional profiles of food products, thereby helping consumers to make informed food choices at the point of purchase. We consider that a meal index such as the MBI, applied on nutrition data of online culinary recipes, canteen or restaurant menus, could potentially support consumers in understanding the extent to which their meal is balanced and aligned with food-based dietary guidelines; however, real-life consumer testing would be needed to measure its impact on eating behavior. The MBI holds promise as a tool for promoting healthy diet behavior.

## Supporting information

S1 File Supplementary material. Methodology and composition of exemplary menu plans.
(PDF)
