# Supplementary material for: Development and validation of a meal quality index with applications to NHANES 2005-2014
Source: PLoS One. 2020 Dec 22;15(12):e0244391. doi: 10.1371/journal.pone.0244391 (PMC7755194; doi:10.1371/journal.pone.0244391)
Supplement: S1 File — Methodology and composition of exemplary menu plans. (PDF) [file pone.0244391.s001.pdf]

SUPPLEMENTARY MATERIAL

Methodology for the development of menu plans

A 2-week menu plan was developed for each of the following three eating patterns of the 2015-2020 DGA, the healthy US, VEG and MED. A 1-week menu plan was developed based on the 2015 Mexican dietary guidelines. The task of completing these menu plans to align with daily and weekly national guidelines and the constraints of the key requirements and limitations were accomplished with the following steps (

Figure 1). The selection of food and beverage items were determined by key healthy eating recommendations from national guidelines e.g. when aligning with the recommendation for dairy items, low-fat milk was chosen for inclusion where appropriate. Generic food and beverage items were selected. Additionally, the following items were selected as fortified when available and appropriate to fit all other requirements; milk, chocolate milk, yogurt, margarine, orange juice, apple juice and grape juice. Food and beverage items containing saturated fats, added sugars, added sodium or alcohol were limited to meet national guidelines.

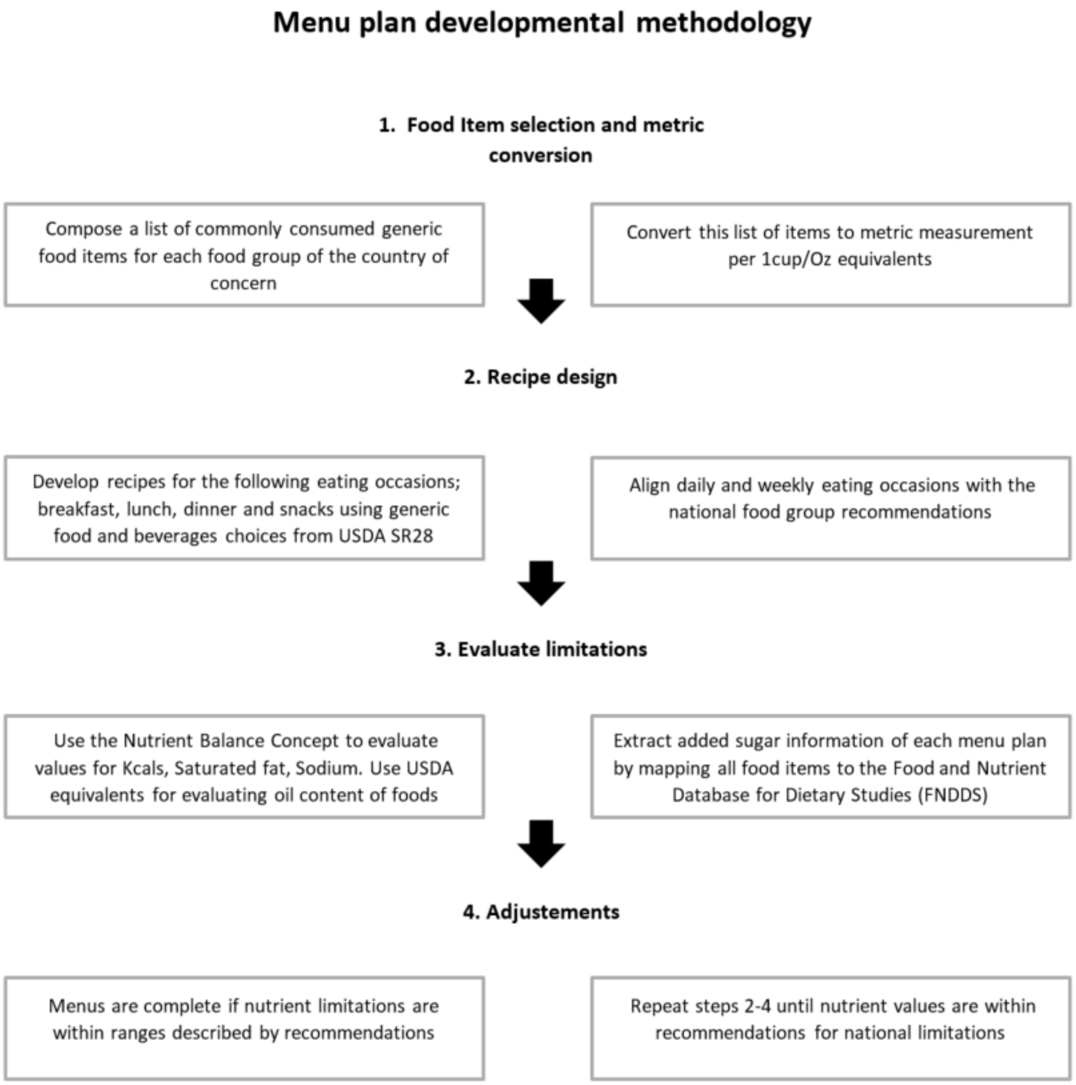

Figure 1: 1. Step-by-step methodology

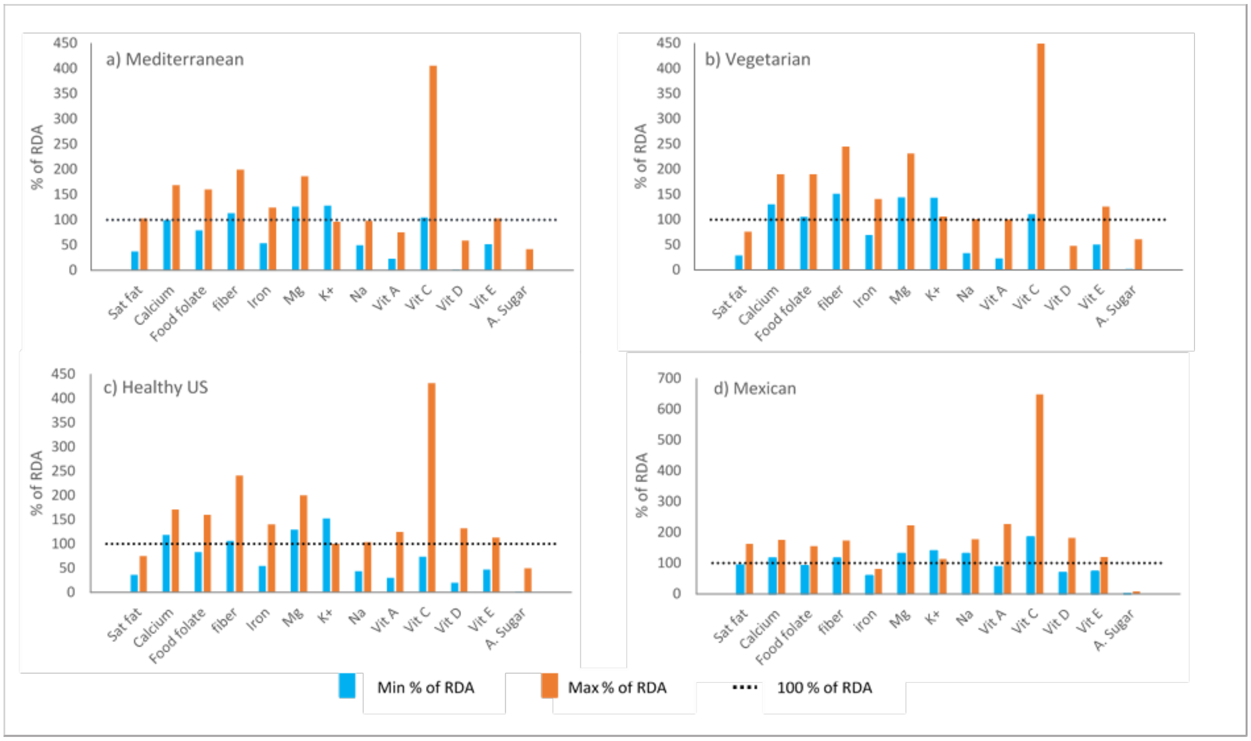

Figure S2. Nutrient profile of each menu plans expressed as a percentage of the national RDA for a 40 year old non-pregnant, non-lactating woman for a) Mediterranean, b) Vegetarian, c) US and d) Mexican.

Mediterranean menu plan

Day	Meal	Usda	Item	grams
1	Breakfast	8122	Cereals, oats, instant, fortified, plain, dry	28
1	Breakfast	9316	Strawberries, raw, sliced	83
1	Breakfast	1256	Yogurt, Greek, plain, nonfat (170)	85
1	Breakfast	12063	Nuts, almonds, dry roasted, without salt added (11 whole kernels)	14.2
1	Breakfast	1129	Egg, whole, cooked, hard-boiled, 1 large egg	50
1	Breakfast	1129	Egg, whole, cooked, hard-boiled, 1 large egg	50
1	Lunch	18076	Bread, whole-wheat, commercially prepared, toasted	50
1	Lunch	15121	Fish, tuna, light, canned in water, drained solids	113.2

continued on next page

continued from previous page

1	Lunch	11168	Corn, sweet, yellow, cooked, boiled, drained, without salt,1 ear small (5-1/2" to 6-1/2" long)	44.5
1	Lunch	11251	Lettuce, cos or romaine, raw, shredded (94)	47
1	Lunch	43355	Mayonnaise, low sodium, low calorie or diet	21
1	Lunch	16229	Soymilk (All flavors), lowfat, with added calcium, vitamins A and D	243
1	Dinner	20029	Couscous, cooked	157.6
1	Dinner	4053	Oil, olive, salad or cooking	13.5
1	Dinner	11032	Lima beans, immature seeds, cooked, boiled, drained, without salt	42.5
1	Dinner	16043	Beans, pinto, mature seeds, cooked, boiled, without salt (171)	85.5
1	Dinner	11529	Tomatoes, red, ripe, raw, year round average, 1 large whole (3" diameter) (123)	61.5
1	Dinner	11333	Peppers, sweet, green, raw,chopped	74.5
1	Dinner	9195	Olives, pickled, canned or bottled, green, 1 olive	10.8
1	Dinner	9252	Pears, raw,1 medium	178
1	Dinner	9040	Bananas, raw, sliced	150
1	Snacks	18216	Crackers, crispbread, rye, 2 crispbread (20)	20
1	Snacks	12195	Nuts, almond butter, plain, without salt added (16)	16
1	Snacks	16158	Hummus, commercial	15
1	Snacks	1082	Milk, lowfat, fluid, 1% milkfat, with added vitamin A and vitamin D (244)	122
1	Snacks	1082	Milk, lowfat, fluid, 1% milkfat, with added vitamin A and vitamin D (244)	122
1	Water	14555	Water, bottled, generic	2000
2	Breakfast	1131	Egg, whole, cooked, poached (1 large)	50
2	Breakfast	18075	Bread, whole-wheat, commercially prepared,1 slice	64
2	Breakfast	9037	Avocados, raw, all commercial varieties, sliced	68
2	Breakfast	1082	Milk, lowfat, fluid, 1% milkfat, with added vitamin A and vitamin D (244)	244
2	Lunch	4053	Oil, olive, salad or cooking	20.25
2	Lunch	15047	Fish, mackerel, Atlantic, cooked, dry heat, (28.3)	84.9
2	Lunch	20037	Rice, brown, long-grain, cooked	101

continued on next page

continued from previous page

2	Lunch	11510	Sweet potato, cooked, boiled, without skin, mashed	164
2	Lunch	11019	Asparagus, frozen, cooked, boiled, drained, without salt (180)	45
2	Lunch	11053	Beans, snap, green, cooked, boiled, drained, without salt (125)	62.5
2	Dinner	15165	Mollusks, mussel, blue, cooked, moist heat	56.6
2	Dinner	20125	Pasta, whole-wheat, cooked , spaghetti packed	151
2	Dinner	18036	Bread, multi-grain, toasted (includes whole-grain)	24
2	Dinner	11885	Tomatoes, red, ripe, canned, packed in tomato juice, no salt added (240)	120
2	Dinner	11457	Spinach,raw	15
2	Dinner	9266	Pineapple, raw, all varieties, chunks	165
2	Dinner	9200	Oranges, raw, all commercial varieties, 2 small (2-3/8" diameter)	48
2	Snacks	9003	Apples, raw, with skin, slices	109
2	Snacks	1220	Yogurt, vanilla, low fat, 11 grams protein per 8 ounce, fortified with vitamin D (245)	245
2	Snacks	12155	Nuts, walnuts, english, 7 halves	7.1
2	Snacks	12155	Nuts, walnuts, english,	7.1
2	Water	14555	Water, bottled, generic	2000
3	Breakfast	1256	Yogurt, Greek, plain, nonfat (170)	170
3	Breakfast	12037	Seeds, sunflower seed kernels, dry roasted, without salt	7.1
3	Breakfast	9054	Blueberries, frozen, unsweetened, unthawed	155
3	Breakfast	1082	Milk, lowfat, fluid, 1% milkfat, with added vitamin A and vitamin D (244)	244
3	Breakfast	8123	Cereals, oats, instant, fortified, plain, prepared with water (boiling water added or microwaved) (117)	117
3	Lunch	20125	Pasta, whole-wheat, cooked , penne	97
3	Lunch	9195	Olives, pickled, canned or bottled, green, 1 olive	10.8
3	Lunch	11529	Tomatoes, red, ripe, raw, year round average,	37.2
3	Lunch	11032	Lima beans, immature seeds, cooked, boiled, drained, without salt (170)	85
3	Lunch	7933	Chicken breast, oven-roasted, fat-free, sliced (28.3)	84.3
3	Lunch	4053	Oil, olive, salad or cooking	6.75

continued on next page

continued from previous page

3	Dinner	16070	Lentils, mature seeds, cooked, boiled, without salt	49.59
3	Dinner	20137	Quinoa, cooked	185
3	Dinner	15137	Crustaceans, crab, alaska king, cooked, moist heat	56.6
3	Dinner	11305	Peas, green, cooked, boiled, drained, without salt (160)	80
3	Dinner	16057	Chickpeas (garbanzo beans, bengal gram), mature seeds, cooked, boiled, without salt	41
3	Dinner	11885	Tomatoes, red, ripe, canned, packed in tomato juice, no salt added	120
3	Dinner	11821	Peppers, sweet, red, raw,1 Large, approx 3-3/4" long, 3" diameter	82
3	Dinner	4053	Oil, olive, salad or cooking	13.5
3	Dinner	9326	Watermelon, raw, diced	76
3	Dinner	9003	Apple, raw, with skin, 1 small small (2-3/4" diameter) (149)	149
3	Snacks	18216	Crackers, crispbread, rye, 2 crispbread	20
3	Snacks	9279	Plums, raw, sliced	165
3	Snacks	9040	Bananas, raw, 1 large (8" to 8-7/8" long) (136)	136
3	Snacks	1082	Milk, lowfat, fluid, 1% milkfat, with added vitamin A and vitamin D (244)	122
3	Water	14555	Water, bottled, generic	2000
4	Breakfast	1129	Egg, whole, cooked, hard-boiled, 1 large egg	100
4	Breakfast	18076	Bread, whole-wheat, commercially prepared, toasted, 1 slice	50
4	Breakfast	11529	Tomatoes, red, ripe, raw, year round average, cherry tomatoes	74.4
4	Breakfast	1082	Milk, lowfat, fluid, 1% milkfat, with added vitamin A and vitamin D (244)	244
4	Lunch	28318	Bread, french or vienna, whole wheat,1 slice	96
4	Lunch	6192	Split pea soup, canned, reduced sodium, prepared with water or ready-to serve	126.5
4	Lunch	1243	Fat free ice cream, no sugar added, flavors other than chocolate	93
4	Lunch	9286	Pomegranates, raw	43.5
4	Dinner	20137	Quinoa, cooked	185
4	Dinner	11053	Beans, snap, green, cooked, boiled, drained, without salt	62.5
4	Dinner	11206	Cucumber, peeled, raw,sliced	59.5

continued on next page

continued from previous page

4	Dinner	11032	Lima beans, immature seeds, cooked, boiled, drained, without salt	85
4	Dinner	11885	Tomatoes, red, ripe, canned, packed in tomato juice, no salt added (240)	120
4	Dinner	7933	Chicken breast, oven-roasted, fat-free, sliced	99.05
4	Dinner	4053	Oil, olive, salad or cooking	27
4	Snacks	1220	Yogurt, vanilla, low fat, 11 grams protein per 8 ounce, fortified with vitamin D (245)	245
4	Snacks	9111	Grapefruit, raw, pink and red and white, all areas, sections	230
4	Snacks	9210	Orange juice, chilled, includes from concentrate, with added calcium and vitamin D (249)	249
4	Snacks	9181	Melons, cantaloupe, raw, balls	78
4	Water	14555	Water, bottled, generic	2000
5	Breakfast	1129	Egg, whole, cooked, hard-boiled, 1 large egg	100
5	Breakfast	1168	Cheese, low fat, cheddar or colby, shredded	18.85
5	Breakfast	4053	Oil, olive, salad or cooking	13.5
5	Breakfast	11529	Tomatoes, red, ripe, raw, year round average, 2 small whole (2-2/5" diameter)	91
5	Breakfast	11457	Spinach,raw	15
5	Lunch	18042	Bread, pita, whole-wheat,1 small (4" diameter) (28)	56
5	Lunch	11252	Lettuce, iceberg (includes crisphead types), raw, chopped 1/2" pieces, loosely packed (114)	28.5
5	Lunch	5168	Turkey, whole, meat only, cooked, roasted (28.3)	70.75
5	Lunch	9195	Olives, pickled, canned or bottled, green, 1 olive (2.7 (0.41g oil)	10.8
5	Dinner	20029	Couscous, cooked	157.6
5	Dinner	15116	Fish, trout, rainbow, wild, cooked, dry heat	56.6
5	Dinner	11099	Brussels sprouts, cooked, boiled, drained, without salt	78
5	Dinner	11934	Potatoes , mashed, home-prepared, whole milk and butter added -(Recipe: Potatoes, 80% ; whole milk, 14% ; butter, 5% ; and salt, 1%)	105
5	Dinner	16057	Chickpeas (garbanzo beans, bengal gram), mature seeds, cooked, boiled, without salt	82
5	Dinner	4053	Oil, olive, salad or cooking	6.25
5	Dinner	1243	Fat free ice cream, no sugar added, flavors other than chocolate	93
5	Dinner	9236	Peaches, yellow, raw, Slices	175

continued on next page

continued from previous page

5	Snacks	8120	Cereals, oats, regular and quick, not fortified, dry	27
5	Snacks	1220	Yogurt, vanilla, low fat, 11 grams protein per 8 ounce, fortified with vitamin D (245)	245
5	Snacks	9040	Bananas, raw, sliced (150)	150
5	Snacks	9298	Raisins, seedless, packed (82.5)	41.25
5	Snacks	18216	Crackers, crispbread, rye, 2 crispbread (20)	20
5	Snacks	9252	Pears, raw,1 medium (178)	178
5	Water	14555	Water, bottled, generic	2000
6	Breakfast	1129	Egg, whole, cooked, hard-boiled, 1 large egg	50
6	Breakfast	1168	Cheese, low fat, cheddar or colby (42.5)	21.25
6	Breakfast	18036	Bread, multi-grain, toasted (includes whole-grain)	48
6	Breakfast	9210	Orange juice, chilled, includes from concentrate, with added calcium and vitamin D (249)	249
6	Lunch	7933	Chicken breast, oven-roasted, fat-free, sliced	56.6
6	Lunch	11885	Tomatoes, red, ripe, canned, packed in tomato juice, no salt added (240)	60
6	Lunch	11261	Mushrooms, white, cooked, boiled, drained, without salt (156)	39
6	Lunch	20037	Rice, brown, long-grain, cooked (101)	101
6	Lunch	16057	Chickpeas (garbanzo beans, bengal gram), mature seeds, cooked, boiled, without salt	41
6	Lunch	1168	Cheese, low fat, cheddar or colby, shredded	18.85
6	Lunch	11168	Corn, sweet, yellow, cooked, boiled, drained, without salt, cut (149)	37.25
6	Dinner	20125	Pasta, whole-wheat, cooked , shells	105
6	Dinner	11053	Beans, snap, green, cooked, boiled, drained, without salt	62.5
6	Dinner	11885	Tomatoes, red, ripe, canned, packed in tomato juice, no salt added	120
6	Dinner	11313	Peas, green, frozen, cooked, boiled, drained, without salt (80)	40
6	Dinner	23478	Beef, ground, 97% lean meat / 3% fat, patty, cooked, broiled	99.05
6	Dinner	4053	Oil, olive, salad or cooking	27
6	Dinner	9132	Grapes,32 red or green (European type, such as Thompson seedless), raw	78.4
6	Snacks	9040	Bananas, raw, sliced (150)	150

continued on next page

continued from previous page

6	Snacks	1220	Yogurt, vanilla, low fat, 11 grams protein per 8 ounce, fortified with vitamin D (245)	245
6	Snacks	18061	Bread, rye, toasted, regular slice (24)	24
6	Snacks	1129	Egg, whole, cooked, hard-boiled, 1 large egg	50
6	Snacks	1082	Milk, lowfat, fluid, 1% milkfat, with added vitamin A and vitamin D (244)	244
6	Water	14555	Water, bottled, generic	2000
7	Breakfast	11529	Tomatoes, red, ripe, raw, year round average, cherry tomatoes	37.25
7	Breakfast	11091	Broccoli, cooked, boiled, drained, without salt, chopped (156)	39
7	Breakfast	15209	Fish, salmon, Atlantic, wild, cooked, dry heat	56.6
7	Breakfast	1131	Egg, whole, cooked, poached (1 large) (50)	100
7	Breakfast	4053	Oil, olive, salad or cooking	13.5
7	Lunch	20029	Couscous, cooked (78.5)	157.6
7	Lunch	9195	Olives, pickled, canned or bottled, green, 1 olive (2.7 (0.41g oil)	21.6
7	Lunch	1029	Cheese, mozzarella, low moisture, part-skim, shredded (28.7)	28.7
7	Lunch	11168	Corn, sweet, yellow, cooked, boiled, drained, without salt, cut (149)	111.75
7	Lunch	11529	Tomatoes, red, ripe, raw, year round average, 1 large whole (3" diameter) (123)	61.5
7	Dinner	10011	Pork, fresh, leg (ham), whole, separable lean only, cooked, roasted (28.3)	70.75
7	Dinner	20037	Rice, brown, long-grain, cooked (101)	101
7	Dinner	9278	Plantains, cooked, sliced (154)	77
7	Dinner	11313	Peas, green, frozen, cooked, boiled, drained, without salt (80)	40
7	Dinner	4053	Oil, olive, salad or cooking	6.25
7	Dinner	9050	Blueberries, raw (148)	74
7	Dinner	9040	Bananas, raw, 1 large (8" to 8-7/8" long) (136)	136
7	Snacks	1082	Milk, lowfat, fluid, 1% milkfat, with added vitamin A and vitamin D (244)	244
7	Snacks	18061	Bread, rye, toasted, regular slice (24)	48
7	Snacks	9094	Figs, dried, uncooked (74.5)	37.25
7	Snacks	18216	Crackers, crispbread, rye, 2 crispbread (20)	20

continued on next page

continued from previous page

7	Snacks	12195	Nuts, almond butter, plain, without salt added (16)	16
7	Snacks	1220	Yogurt, vanilla, low fat, 11 grams protein per 8 ounce, fortified with vitamin D (245)	122.5
7	Water	14555	Water, bottled, generic	2000
8	Breakfast	8123	Cereals, oats, instant, fortified, plain, prepared with water (boiling water added or microwaved) (117)	117
8	Breakfast	1082	Milk, lowfat, fluid, 1% milkfat, with added vitamin A and vitamin D (244)	244
8	Breakfast	12016	Seeds, pumpkin and squash seed kernels, roasted, without salt (14.2)	7.1
8	Breakfast	12155	Nuts, walnuts, english, 7 halves (14.2)	7.1
8	Breakfast	9040	Bananas, raw, sliced (150)	150
8	Breakfast	18076	Bread, whole-wheat, commercially prepared, toasted, 1 slice, (25)	50
8	Breakfast	1129	Egg, whole, cooked, hard-boiled, 1 large egg	50
8	Lunch	20137	Quinoa, cooked (92.5)	157
8	Lunch	11291	Onions, spring or scallions (includes tops and bulb), raw, chopped (100)	52.75
8	Lunch	11529	Tomatoes, red, ripe, raw, year round average, cherry tomatoes (149)	111.75
8	Lunch	7933	Chicken breast, oven-roasted, fat-free, sliced (28.3)	70.75
8	Lunch	4053	Oil, olive, salad or cooking	6.25
8	Lunch	9195	Olives, pickled, canned or bottled, green, 1 olive	8.1
8	Lunch	11053	Beans, snap, green, cooked, boiled, drained, without salt (125)	62.5
8	Dinner	11367	Potatoes, boiled, cooked without skin, flesh, without salt (156)	156
8	Dinner	4053	Oil, olive, salad or cooking	13.5
8	Dinner	11529	Tomatoes, red, ripe, raw, year round average, 2 small whole (2-2/5" diameter) (182)	91
8	Dinner	15034	Fish, haddock, cooked, dry heat (28.3)	84.9
8	Dinner	9195	Olives, pickled, canned or bottled, green, 1 olive	8.1
8	Dinner	11019	Asparagus, frozen, cooked, boiled, drained, without salt (180)	45
8	Dinner	9316	Strawberries, raw, 8 large (1-3/8" diameter), (144)	72
8	Dinner	1220	Yogurt, vanilla, low fat, 11 grams protein per 8 ounce, fortified with vitamin D (245)	245
8	Snacks	9210	Orange juice, chilled, includes from concentrate, with added calcium and vitamin D (249)	249

continued on next page

continued from previous page

8	Snacks	18235	Crackers, whole-wheat, 5 crackers (23)	23
8	Snacks	12195	Nuts, almond butter, plain, without salt added (16)	8
8	Water	14555	Water, bottled, generic	2000
9	Breakfast	1131	Egg, whole, cooked, poached (1 large) (50)	100
9	Breakfast	1082	Milk, lowfat, fluid, 1% milkfat, with added vitamin A and vitamin D (244)	244
9	Breakfast	1019	Cheese, feta, crumbled (150)	37.5
9	Breakfast	11529	Tomatoes, red, ripe, raw, year round average, 1 large whole (3" diameter) (123)	30.75
9	Breakfast	28397	Bread, multi-grain (includes whole-grain),1 slice regular (26)	52
9	Lunch	15151	Crustaceans, shrimp, mixed species, cooked, moist heat (may have been previously frozen)	70.75
9	Lunch	20125	Pasta, whole-wheat, cooked , spaghetti packed (75.5)	226.5
9	Lunch	11458	Spinach, cooked, boiled, drained, without salt (180)	45
9	Lunch	4053	Oil, olive, salad or cooking	6.25
9	Lunch	18076	Bread, whole-wheat, commercially prepared, toasted, 1 slice, (25)	25
9	Lunch	1032	Cheese, parmesan, grated (42.5)	10.6
9	Dinner	11357	Potatoes, white, flesh and skin, baked, medium (2-1/4" to 3-1/4" diameter) (173)	173
9	Dinner	11478	Squash, summer, zucchini, includes skin, cooked, boiled, drained, without salt, sliced (180)	45
9	Dinner	11283	Onions, cooked, boiled, drained, without salt (210)	52.5
9	Dinner	11821	Peppers, sweet, red, raw,1 Large, approx 3-3/4" long, 3" diameter (164)	82
9	Dinner	7933	Chicken breast, oven-roasted, fat-free, sliced (28.3)	56.6
9	Dinner	4053	Oil, olive, salad or cooking	13.5
9	Dinner	11529	Tomatoes, red, ripe, raw, year round average, 2 small whole (2-2/5" diameter) (182)	91
9	Dinner	1220	Yogurt, vanilla, low fat, 11 grams protein per 8 ounce, fortified with vitamin D (245)	122.5
9	Dinner	9003	Apple, raw, with skin, 1 small small (2-3/4" diameter) (149)	149
9	Snacks	9200	Oranges, raw, all commercial varieties, 1 large (3-1/16" diameter) (184)	184
9	Snacks	9326	Watermelon, raw, 12 balls (146.4)	73.2
9	Snacks	1220	Yogurt, vanilla, low fat, 11 grams protein per 8 ounce, fortified with vitamin D (245)	122.5

continued on next page

continued from previous page

9	Snacks	12063	Nuts, almonds, dry roasted, without salt added (11 whole kernels) (14.2)	14.2
9	Water	14555	Water, bottled, generic	2000
10	Breakfast	8123	Cereals, oats, instant, fortified, plain, prepared with water (boiling water added or microwaved) (117)	117
10	Breakfast	12037	Seeds, sunflower seed kernels, dry roasted, without salt (14.2)	7.1
10	Breakfast	1256	Yogurt, Greek, plain, nonfat (170)	85
10	Breakfast	9302	Raspberries, raw (120)	60
10	Breakfast	9040	Bananas, raw, sliced (150)	150
10	Lunch	28397	Bread, multi-grain (includes whole-grain),1 slice regular (26)	52
10	Lunch	15121	Fish, tuna, light, canned in water, drained solids (28.3)	56.6
10	Lunch	43355	Mayonnaise, low sodium, low calorie or diet	28
10	Lunch	11529	Tomatoes, red, ripe, raw, year round average, 1 large whole (3" diameter) (123)	30.75
10	Lunch	11168	Corn, sweet, yellow, cooked, boiled, drained, without salt, cut (149)	73
10	Lunch	11478	Squash, summer, zucchini, includes skin, cooked, boiled, drained, without salt, sliced (180)	90
10	Lunch	1082	Milk, lowfat, fluid, 1% milkfat, with added vitamin A and vitamin D (244)	122
10	Dinner	15047	Fish, mackerel, Atlantic, cooked, dry heat, (28.3)	84.9
10	Dinner	11934	Potatoes , mashed, home-prepared, whole milk and butter added -(Recipe: Potatoes, 80% ; whole milk, 14% ; butter, 5% ; and salt, 1%) (210)	105
10	Dinner	11019	Asparagus, frozen, cooked, boiled, drained, without salt (180)	45
10	Dinner	20037	Rice, brown, long-grain, cooked (101)	303
10	Dinner	4053	Oil, olive, salad or cooking	6.25
10	Dinner	11053	Beans, snap, green, cooked, boiled, drained, without salt (125)	62.5
10	Dinner	9132	Grapes,32 red or green (European type, such as Thompson seedless), raw (156.8)	78.4
10	Dinner	9266	Pineapple, raw, all varieties, chunks (165)	165
10	Snacks	9094	Figs, dried, uncooked (74.5)	37.25
10	Snacks	12155	Nuts, walnuts, english, 7 halves (14.2)	7.1
10	Snacks	1220	Yogurt, vanilla, low fat, 11 grams protein per 8 ounce, fortified with vitamin D (245)	245

continued on next page

continued from previous page

10	Water	14555	Water, bottled, generic	2000
11	Breakfast	9037	Avocados, raw, all commercial varieties, sliced (146)	73
11	Breakfast	18061	Bread, rye, toasted, regular slice (24)	48
11	Breakfast	1019	Cheese, feta, crumbled (150)	75
11	Breakfast	1131	Egg, whole, cooked, poached (1 large) (50)	50
11	Breakfast	1131	Egg, whole, cooked, poached (1 large) (50)	50
11	Breakfast	9210	Orange juice, chilled, includes from concentrate, with added calcium and vitamin D (249)	249
11	Lunch	28397	Bread, multi-grain (includes whole-grain),1 slice regular	52
11	Lunch	11251	Lettuce, cos or romaine, raw, shredded (94)	47
11	Lunch	11283	Onions, cooked, boiled, drained, without salt (210)	52.5
11	Lunch	17012	Lamb, domestic, leg, whole (shank and sirloin), separable lean and fat, trimmed to 1/4" fat, choice, cooked, roasted	70.75
11	Lunch	11206	Cucumber, peeled, raw,sliced (119)	29.75
11	Lunch	4017	Salad dressing, thousand island, commercial, regular	28
11	Lunch	1082	Milk, lowfat, fluid, 1% milkfat, with added vitamin A and vitamin D (244)	246
11	Dinner	15209	Fish, salmon, Atlantic, wild, cooked, dry heat	84.9
11	Dinner	11821	Peppers, sweet, red, raw,1 Large, approx 3-3/4" long, 3" diameter (164)	82
11	Dinner	11261	Mushrooms, white, cooked, boiled, drained, without salt (156)	78
11	Dinner	20125	Pasta, whole-wheat, cooked , spaghetti packed (75.5)	151
11	Dinner	4053	Oil, olive, salad or cooking	13.5
11	Dinner	9132	Grapes,32 red or green (European type, such as Thompson seedless), raw (156.8)	78.4
11	Dinner	1256	Yogurt, Greek, plain, nonfat (170)	85
11	Snacks	9050	Blueberries, raw (148)	148
11	Snacks	9003	Apple, raw, with skin, 1 small small (2-3/4" diameter) (149)	149
11	Snacks	9286	Pomegranates, raw	87
11	Water	14555	Water, bottled, generic	2000
12	Breakfast	28397	Bread, multi-grain (includes whole-grain),1 slice regular (26)	52

continued on next page

continued from previous page

12	Breakfast	11529	Tomatoes, red, ripe, raw, year round average, 1 large whole (3" diameter) (123)	99.25
12	Breakfast	1131	Egg, whole, cooked, poached (1 large) (50)	100
12	Breakfast	16229	Soymilk (All flavors), lowfat, with added calcium, vitamins A and D (243)	243
12	Lunch	18061	Bread, rye, toasted, regular slice (24)	48
12	Lunch	16028	Beans, kidney, all types, mature seeds, cooked, boiled, without salt (44.25)	22.13
12	Lunch	11251	Lettuce, cos or romaine, raw, shredded (94)	23.5
12	Lunch	7933	Chicken breast, oven-roasted, fat-free, sliced (28.3)	70.75
12	Lunch	43355	Mayonnaise, low sodium, low calorie or diet	21
12	Lunch	6192	Split pea soup, canned, reduced sodium, prepared with water or ready-to serve (126.5)	189.75
12	Dinner	20137	Quinoa, cooked (92.5)	185
12	Dinner	16028	Beans, kidney, all types, mature seeds, cooked, boiled, without salt (177)	177
12	Dinner	11032	Lima beans, immature seeds, cooked, boiled, drained, without salt (170)	85
12	Dinner	4053	Oil, olive, salad or cooking	20.25
12	Dinner	11529	Tomatoes, red, ripe, raw, year round average, 1 large whole (3" diameter) (123)	61.5
12	Dinner	11283	Onions, cooked, boiled, drained, without salt (210)	52.5
12	Dinner	9003	Apple, raw, with skin, 1 small small (2-3/4" diameter) (149)	149
12	Snacks	9316	Strawberries, raw, sliced (166)	83
12	Snacks	9111	Grapefruit, raw, pink and red and white, all areas, sections (230)	230
12	Snacks	1256	Yogurt, Greek, plain, nonfat (170)	170
12	Snacks	9040	Bananas, raw, 1 large (8" to 8-7/8" long) (136)	136
12	Water	14555	Water, bottled, generic	2000
13	Breakfast	1256	Yogurt, Greek, plain, nonfat (170)	170
13	Breakfast	8122	Cereals, oats, instant, fortified, plain, dry, 1 packet (28)	28
13	Breakfast	9050	Blueberries, raw (148)	74
13	Breakfast	9040	Bananas, raw, 2 small ,less than 6" long (81)	40.5
13	Breakfast	16229	Soymilk (All flavors), lowfat, with added calcium, vitamins A and D (243)	121.5

continued on next page

continued from previous page

13	Lunch	23478	Beef, ground, 97% lean meat / 3% fat, patty, cooked, broiled (28.3)	99.05
13	Lunch	20037	Rice, brown, long-grain, cooked (101)	202
13	Lunch	11053	Beans, snap, green, cooked, boiled, drained, without salt (125)	62.5
13	Lunch	16028	Beans, kidney, all types, mature seeds, cooked, boiled, without salt (177)	88.5
13	Lunch	11510	Sweet potato, cooked, boiled, without skin, mashed (328)	246
13	Lunch	4053	Oil, olive, salad or cooking	13.5
13	Dinner	7933	Chicken breast, oven-roasted, fat-free, sliced (28.3)	84.9
13	Dinner	20125	Pasta, whole-wheat, cooked , spaghetti packed (75.5)	226.5
13	Dinner	1032	Cheese, parmesan, grated (42.5)	28.35
13	Dinner	11529	Tomatoes, red, ripe, raw, year round average, 1 large whole (3" diameter) (123)	61.5
13	Dinner	4053	Oil, olive, salad or cooking	13.5
13	Dinner	11313	Peas, green, frozen, cooked, boiled, drained, without salt (80)	20
13	Dinner	9252	Pears, raw, slices (140)	140
13	Snacks	9316	Strawberries, raw, sliced (166)	83
13	Snacks	18235	Crackers, whole-wheat, 5 crackers (23)	23
13	Water	14555	Water, bottled, generic	2000
14	Breakfast	18061	Bread, rye, toasted, regular slice (24)	48
14	Breakfast	15209	Fish, salmon, Atlantic, wild, cooked, dry heat	42.45
14	Breakfast	1131	Egg, whole, cooked, poached (1 large) (50)	50
14	Breakfast	9210	Orange juice, chilled, includes from concentrate, with added calcium and vitamin D (249)	249
14	Lunch	20029	Couscous, cooked (78.5)	157.6
14	Lunch	7933	Chicken breast, oven-roasted, fat-free, sliced (28.3)	56.5
14	Lunch	11821	Peppers, sweet, red, raw, chopped (149)	41
14	Lunch	11529	Tomatoes, red, ripe, raw, year round average, 1 large whole (3" diameter) (123)	61.5
14	Lunch	11168	Corn, sweet, yellow, cooked, boiled, drained, without salt, 1 ear large (7-3/4" to 9" long) (118)	88.5
14	Lunch	11283	Onions, cooked, boiled, drained, without salt (210)	52.5

continued on next page

continued from previous page

14	Lunch	9195	Olives, pickled, canned or bottled, green, 1 olive	16.2
14	Dinner	10011	Pork, fresh, leg (ham), whole, separable lean only, cooked, roasted (28.3)	56.6
14	Dinner	11091	Broccoli, cooked, boiled, drained, without salt, chopped (156)	39
14	Dinner	9278	Plantains, cooked, sliced (154)	77
14	Dinner	20037	Rice, brown, long-grain, cooked (101)	202
14	Dinner	4053	Oil, olive, salad or cooking	20.25
14	Dinner	1082	Milk, lowfat, fluid, 1% milkfat, with added vitamin A and vitamin D (244)	122
14	Dinner	9003	Apple, raw, with skin, 1 small small (2-3/4" diameter) (149)	149
14	Snacks	9298	Raisins, seedless, packed (82.5)	41.25
14	Snacks	9302	Raspberries, raw (120)	120
14	Snacks	1220	Yogurt, vanilla, low fat, 11 grams protein per 8 ounce, fortified with vitamin D (245)	245
14	Snacks	1082	Milk, lowfat, fluid, 1% milkfat, with added vitamin A and vitamin D (244)	244
14	Water	14555	Water, bottled, generic	2000

Mexican-style menu plan

**Day**	**Meal**	**Usda**	**Item**	**Grams**
**1**	**Breakfast**	14209	Beverages, coffee, brewed, prepared with tap water	180
		08402	Cereals, Quick Oats, Dry	60
		01175	Milk, fluid, 1% fat, without added vitamin A and vitamin D	240
		09226	Papayas, raw	145
	**Lunch**	11091	Broccoli, cooked, boiled, drained, without salt	78
		16057	Chickpeas (garbanzo beans, bengal gram), mature seeds, cooked, boiled, without salt	100
		11210	Eggplant, cooked, boiled, drained, without salt	75
		15261	Fish, tilapia, raw	90

continued on next page

continued from previous page

		04053	Oil, olive, salad or cooking	10
		20451	Rice, white, medium-grain, cooked, unenriched	90
		18363	Tortillas, ready-to-bake or -fry, corn	30
	**Dinner**	11967	Cauliflower, green, cooked, no salt added	95
		07961	Chicken breast, deli, rotisserie seasoned, sliced, prepackaged	75
		04053	Oil, olive, salad or cooking	10
		09206	Orange juice, raw	160
		11364	Potatoes, baked, skin, without salt	140
	**Snack**	08144	Cereals, whole wheat hot natural cereal, dry	25
		09316	Strawberries, raw	160
		01116	Yogurt, plain, whole milk, 8 grams protein per 8 ounce	180
		12061	Nuts, almonds	12
		09252	Pears, raw	95
	**Breakfast**	14209	Beverages, coffee, brewed, prepared with tap water	100
**2**		18967	Bread, white wheat	60
		43344	Jams, preserves, marmalade, reduced sugar	30
		09148	Kiwifruit, green, raw	115
		01175	Milk, fluid, 1% fat, without added vitamin A and vitamin D	240
	**Lunch**	23220	Beef, ground, unspecified fat content, cooked	80
		11124	Carrots, raw	55
		16370	Lentils, mature seeds, cooked, boiled, with salt	100
		04053	Oil, olive, salad or cooking	15
		20447	Rice, white, long-grain, parboiled, unenriched, cooked	100
		11695	Tomatoes, orange, raw	60
	**Dinner**	07961	Chicken breast, deli, rotisserie seasoned, sliced, prepackaged	25
		11908	Corn, sweet, white, canned, vacuum pack, regular pack	20

continued on next page

continued from previous page

		16173	Frijoles rojos volteados (Refried beans, red, canned)	75
		11253	Lettuce, green leaf, raw	70
		04053	Oil, olive, salad or cooking	10
		11695	Tomatoes, orange, raw	90
		28295	Tortillas, ready-to-bake or -fry, whole wheat	30
	**Snacks**	09050	Blueberries, raw	100
		01116	Yogurt, plain, whole milk, 8 grams protein per 8 ounce	180
		18065	Bread, wheat, toasted	60
		01036	Cheese, ricotta, whole milk	60
		09206	Orange juice, raw	80
	**Breakfast**	14209	Beverages, coffee, brewed, prepared with tap water	100
		28397	Bread, multi-grain (includes whole-grain)	75
**3**		01132	Egg, whole, cooked, scrambled	100
		09181	Melons, cantaloupe, raw	160
		01211	Milk, whole, 3.25% milkfat, without added vitamin A and vitamin D	240
	**Lunch**	11210	Eggplant, cooked, boiled, drained, without salt	75
		04053	Oil, olive, salad or cooking	15
		11283	Onions, cooked, boiled, drained, without salt	25
		20421	Pasta, cooked, unenriched, without added salt	120
		11477	Squash, summer, zucchini, includes skin, raw	50
		09221	Tangerine juice, raw	160
	**Dinner**	11080	Beets, raw	25
		16370	Lentils, mature seeds, cooked, boiled, with salt	200
		11251	Lettuce, cos or romaine, raw	45
		04053	Oil, olive, salad or cooking	10
		20451	Rice, white, medium-grain, cooked, unenriched	100

continued on next page

continued from previous page

	**Snacks**	09040	Bananas, raw	90
		01211	Milk, whole, 3.25% milkfat, without added vitamin A and vitamin D	240
		20038	Oats	30
		11974	Grape leaves, raw	90
	**Breakfast**	08120	Cereals, oats, regular and quick, not fortified, dry	30
		01211	Milk, whole, 3.25% milkfat, without added vitamin A and vitamin D	240
		12142	Nuts, pecans	15
**4**		09316	Strawberries, raw	100
	**Lunch**	09037	Avocados, raw, all commercial varieties	50
		01009	Cheese, cheddar	20
		07961	Chicken breast, deli, rotisserie seasoned, sliced, prepackaged	35
		16173	Frijoles rojos volteados (Refried beans, red, canned)	80
		11260	Mushrooms, white, raw	100
		11282	Onions, raw	25
		09429	Pineapple, raw, traditional varieties	125
		06164	Sauce, salsa, ready-to-serve	30
		11695	Tomatoes, orange, raw	50
		18363	Tortillas, ready-to-bake or -fry, corn	120
	**Dinner**	16057	Chickpeas (garbanzo beans, bengal gram), mature seeds, cooked, boiled, without salt	82
		11205	Cucumber, with peel, raw	65
		01129	Egg, whole, cooked, hard-boiled	50
		15185	Fish, tuna, white, canned in oil, without salt, drained solids	30
		11252	Lettuce, iceberg (includes crisphead types), raw	70
		09176	Mangos, raw	160
		04053	Oil, olive, salad or cooking	15
	**Snacks**	09252	Pears, raw	95

continued on next page

continued from previous page

		14209	Beverages, coffee, brewed, prepared with tap water	100
		18065	Bread, wheat, toasted	50
		01211	Milk, whole, 3.25% milkfat, without added vitamin A and vitamin D	240
	**Breakfast**	14209	Beverages, coffee, brewed, prepared with tap water	100
		18967	Bread, white wheat	50
		01211	Milk, whole, 3.25% milkfat, without added vitamin A and vitamin D	240
		09226	Papayas, raw	140
**5**	**Lunch**	11052	Beans, snap, green, raw	90
		11125	Carrots, cooked, boiled, drained, without salt	80
		15261	Fish, tilapia, raw	75
		09176	Mangos, raw	165
		04053	Oil, olive, salad or cooking	10
		31036	Potatoes, mashed, ready-to-eat	105
	**Dinner**	16370	Lentils, mature seeds, cooked, boiled, with salt	100
		04053	Oil, olive, salad or cooking	10
		10201	Pork, fresh, loin, center rib (chops), boneless, separable lean only, cooked, broiled	50
		11429	Radishes, raw	130
		20451	Rice, white, medium-grain, cooked, unenriched	90
		11603	Yambean (jicama), raw	60
	**snacks**	08402	Cereals, Quick Oats, Dry	30
		09089	Figs, raw	80
		12006	Seeds, chia seeds, dried	12
		01116	Yogurt, plain, whole milk, 8 grams protein per 8 ounce	170
		18065	Bread, wheat, toasted	50
		01228	Cheese, fresh, queso fresco	40
		09206	Orange juice, raw	160

continued on next page

continued from previous page

	**Breakfast**	18967	Bread, white wheat	50
		43344	Jams, preserves, marmalade, reduced sugar	30
		01211	Milk, whole, 3.25% milkfat, without added vitamin A and vitamin D	240
		09266	Pineapple, raw, all varieties	124
	**Lunch**	16005	Beans, baked, home prepared	180
**6**		18065	Bread, wheat, toasted	60
		01009	Cheese, cheddar	30
		04053	Oil, olive, salad or cooking	10
		11695	Tomatoes, orange, raw	100
	**Dinner**	11125	Carrots, cooked, boiled, drained, without salt	70
		04053	Oil, olive, salad or cooking	5
		20421	Pasta, cooked, unenriched, without added salt	120
		06194	Soup, chicken broth, ready-to-serve	240
		11695	Tomatoes, orange, raw	100
		18363	Tortillas, ready-to-bake or -fry, corn	60
	**Snacks**	09040	Bananas, raw	80
		12142	Nuts, pecans	10
		09148	Kiwifruit, green, raw	130
		01116	Yogurt, plain, whole milk, 8 grams protein per 8 ounce	180
	**Breakfast**	18955	Bread, pan dulce, sweet yeast bread	40
		01175	Milk, fluid, 1% fat, without added vitamin A and vitamin D	200
		09316	Strawberries, raw	200
		01228	Cheese, fresh, queso fresco	10
		09152	Lemon juice, raw	180
		11263	Mushrooms, white, stir-fried	30
**7**		18970	Tortillas, ready-to-bake or -fry, flour, shelf stable	30

continued on next page

	**Lunch**	11052	Beans, snap, green, raw	115
		23220	Beef, ground, unspecified fat content, cooked	50
		11136	Cauliflower, cooked, boiled, drained, without salt	95
		04053	Oil, olive, salad or cooking	10
		09203	Oranges, raw, Florida	240
		20451	Rice, white, medium-grain, cooked, unenriched	110
	**Dinner**	01132	Egg, whole, cooked, scrambled	40
		11251	Lettuce, cos or romaine, raw	55
		04053	Oil, olive, salad or cooking	10
		11367	Potatoes, boiled, cooked without skin, flesh, without salt	90
		11458	Spinach, cooked, boiled, drained, without salt	100
		11695	Tomatoes, orange, raw	50
		28295	Tortillas, ready-to-bake or -fry, whole wheat	20
	**Snacks**	09176	Mangos, raw	170
		12006	Seeds, chia seeds, dried	10
		01231	Yogurt, vanilla flavor, lowfat milk, sweetened with low calorie sweetener	150
		09039	Avocados, raw, Florida	60
		11091	Broccoli, cooked, boiled, drained, without salt	25
		11695	Tomatoes, orange, raw	25
		18363	Tortillas, ready-to-bake or -fry, corn	30

US-style menu plan

Day	Meal	Usda	Item	Grams
1	Breakfast	18076	Bread, whole-wheat, commercially prepared, toasted, 1 slice, (25)	50
		4631	Margarine-like, vegetable oil spread, fat-free, tub (14.6) (0.44)	14.6
		1132	Egg, whole, cooked, scrambled (61)	61
		9206	Orange juice, raw (248)	248
	Lunch	18449	Tortillas, ready-to-bake or -fry, corn, without added salt (approx 6" diameter) (26)	26
		11251	Lettuce, cos or romaine, raw, shredded (94)	23.5
		16015	Beans, black, mature seeds, cooked, boiled, without salt (172)	86
		11517	Sweet potato, frozen, cooked, baked, without salt, cubes (176)	88
		11529	Tomatoes, red, ripe, raw, year round average, cherry tomatoes (149)	74.5
		11821	Peppers, sweet, red, raw, chopped	37.25
		27063	Sauce, enchilada, red, mild, ready to serve (1 cup0224g)	56
		1168	Cheese, low fat, cheddar or colby	21.25
		1235	Yogurt, frozen, flavors not chocolate, nonfat milk, with low-calorie sweetener (136)	136
	Dinner	4053	Oil, olive, salad or cooking	13.5
		15047	Fish, mackerel, Atlantic, cooked, dry heat, (28.3)	84.9
		20037	Rice, brown, long-grain, cooked	151.5
		11305	Peas, green, cooked, boiled, drained, without salt (160)	80
		16028	Beans, kidney, all types, mature seeds, cooked, boiled, without salt (177)	44.25

continued on next page

continued from previous page

		1085	Milk, nonfat, fluid, with added vitamin A and vitamin D (fat free or skim) (245)	245
		9096	Fruit cocktail, (peach and pineapple and pear and grape and cherry), canned, water pack, solids and liquids (237)	237
	Snack	18216	Crackers, crispbread, rye, 2 crispbread (20)	30
		16158	Hummus, commercial	15
		11124	Carrots, raw, strips or slices (122)	61
		1085	Milk, nonfat, fluid, with added vitamin A and vitamin D (fat free or skim) (245)	245
2	Breakfast	8123	Cereals, oats, instant, fortified, plain, prepared with water (boiling water added or microwaved) (117)	175.5
		1085	Milk, nonfat, fluid, with added vitamin A and vitamin D (fat free or skim) (245)	245
		9040	Bananas, raw, sliced (150)	150
		1129	Egg, whole, cooked, hard-boiled, 1 large egg	50
		1129	Egg, whole, cooked, hard-boiled, 1 large egg	50
	Lunch	18449	Tortillas, ready-to-bake or -fry, corn, without added salt (approx 6" diameter) (26)	26
		7933	Chicken breast, oven-roasted, fat-free, sliced (28.3)	42.45
		11251	Lettuce, cos or romaine, raw, shredded (94)	23.5
		11529	Tomatoes, red, ripe, raw, year round average, 1 large whole (3" diameter) (123)	30.75
		1168	Cheese, low fat, cheddar or colby, shredded (37.7)	10.625
		43355	Mayonnaise, low sodium, low calorie or diet	14
		16229	Soymilk (All flavors), lowfat, with added calcium, vitamins A and D (243)	121.5
	Dinner	16070	Lentils, mature seeds, cooked, boiled, without salt	49.59
		20137	Quinoa, cooked	185
		15137	Crustaceans, crab, alaska king, cooked, moist heat	56.6

continued on next page

continued from previous page

		11305	Peas, green, cooked, boiled, drained, without salt (160)	80
		16057	Chickpeas (garbanzo beans, bengal gram), mature seeds, cooked, boiled, without salt	82
		11885	Tomatoes, red, ripe, canned, packed in tomato juice, no salt added	120
		11821	Peppers, sweet, red, raw,1 Large, approx 3-3/4" long, 3" diameter	82
		4053	Oil, olive, salad or cooking	13.5
		9266	Pineapple, raw, all varieties, chunks (165)	82.5
	Snacks	1256	Yogurt, Greek, plain, nonfat (170)	170
		9316	Strawberries, raw, sliced (166)	83
		18429	Crackers, whole-wheat, low salt, 5 crackers (20)	30
		1019	Cheese, feta, crumbled (150)	37.5
		9195	Olives, pickled, canned or bottled, green, 1 olive (2.7g)) (0.41g oil)	10.8
3	Breakfast	18267	English muffins, whole-wheat, 1/2 english muffin toasted	61
		1132	Egg, whole, cooked, scrambled (61)	61
		1132	Egg, whole, cooked, scrambled (61)	61
		43449	Beans, baked, canned, no salt added (253)	126.5
		1085	Milk, nonfat, fluid, with added vitamin A and vitamin D (fat free or skim) (245)	122.5
	Lunch	7933	Chicken breast, oven-roasted, fat-free, sliced (28.3)	70.75
		20037	Rice, brown, long-grain, cooked	151.5
		11053	Beans, snap, green, cooked, boiled, drained, without salt (125)	62.5
		11125	Carrots, cooked, boiled, drained, without salt (156)	39
		11305	Peas, green, cooked, boiled, drained, without salt (160)	80
		9252	Pears, raw,1 medium (178)	178

continued on next page

continued from previous page

	Dinner	23478	Beef, ground, 97% lean meat / 3% fat, patty, cooked, broiled (28.3)	56.6
		4053	Oil, olive, salad or cooking	13.5
		11283	Onions, cooked, boiled, drained, without salt (210)	52.5
		11547	Tomato products, canned, puree, without salt added (250)	62.5
		20125	Pasta, whole-wheat, cooked , spaghetti not packed (58.5)	87.75
		11125	Carrots, cooked, boiled, drained, without salt (156)	39
		1168	Cheese, low fat, cheddar or colby, shredded (37.7)	18.85
		1085	Milk, nonfat, fluid, with added vitamin A and vitamin D (fat free or skim) (245)	245
	Snacks	18429	Crackers, whole-wheat, low salt, 5 crackers (20)	20
		16158	Hummus, commercial	15
		1220	Yogurt, vanilla, low fat, 11 grams protein per 8 ounce, fortified with vitamin D (245)	245
		9040	Bananas, raw, 1 large (8" to 8-7/8" long) (136)	136
4	Breakfast	9037	Avocados, raw, all commercial varieties, sliced (146)	73
		18061	Bread, rye, toasted, regular slice (24)	48
		1168	Cheese, low fat, cheddar or colby (42.5)	10.625
		1131	Egg, whole, cooked, poached (1 large) (50)	50
		1131	Egg, whole, cooked, poached (1 large) (50)	50
		9206	Orange juice, raw (248)	248
	Lunch	7933	Chicken breast, oven-roasted, fat-free, sliced (28.3)	42.45
		20029	Couscous, cooked (78.5)	78.5
		11457	Spinach,raw (60)	15
		11124	Carrots, raw, grated (110)	27.5
		11091	Broccoli, cooked, boiled, drained, without salt, chopped (156)	39
		11179	Corn, sweet, yellow, frozen, kernels cut off cob, boiled, drained, without salt (165)	41.25

continued on next page

continued from previous page

		11206	Cucumber, peeled, raw,sliced (119)	29.75
		9266	Pineapple, raw, all varieties, chunks (165)	82.5
	Dinner	15086	Fish, salmon, sockeye, cooked, dry heat, (28.3)	84.9
		20037	Rice, brown, long-grain, cooked (101)	151.5
		11012	Asparagus, cooked, boiled, drained (180)	45
		11821	Peppers, sweet, red, raw, chopped (149)	37.25
		11283	Onions, cooked, boiled, drained, without salt (210)	52.5
		1243	Fat free ice cream, no sugar added, flavors other than chocolate (204)	204
		9326	Watermelon, raw, diced (152)	76
	Snacks	19806	Snacks, popcorn, air-popped (Unsalted) (24)	36
		1085	Milk, nonfat, fluid, with added vitamin A and vitamin D (fat free or skim) (245)	306.25
		9003	Apple, raw, with skin, 1 small small (2-3/4" diameter) (149)	149
5	Breakfast	1220	Yogurt, vanilla, low fat, 11 grams protein per 8 ounce, fortified with vitamin D (245)	122.5
		9054	Blueberries, frozen, unsweetened, unthawed (155)	38.75
		9040	Bananas, raw, sliced (150)	75
		12037	Seeds, sunflower seed kernels, dry roasted, without salt (14.2)	7.1
		8122	Cereals, oats, instant, fortified, plain, dry, 1 packet (28)	28
		18061	Bread, rye, toasted, regular slice (24)	50
		4631	Margarine-like, vegetable oil spread, fat-free, tub (14.6) (0.44)	14.6
	Lunch	28397	Bread, multi-grain (includes whole-grain),1 slice regular (26)	52
		5168	Turkey, whole, meat only, cooked, roasted (28.3)	56.6
		5168	Turkey, whole, meat only, cooked, roasted (28.3)	14.15
		11251	Lettuce, cos or romaine, raw, shredded (94)	23.5
		11168	Corn, sweet, yellow, cooked, boiled, drained, without salt, cut (149)	74.5

continued on next page

continued from previous page

		11529	Tomatoes, red, ripe, raw, year round average, cherry tomatoes (149)	37.25
		1168	Cheese, low fat, cheddar or colby (42.5)	10.625
		43355	Mayonnaise, low sodium, low calorie or diet	14
		1085	Milk, nonfat, fluid, with added vitamin A and vitamin D (fat free or skim) (245)	306.25
		9096	Fruit cocktail, (peach and pineapple and pear and grape and cherry), canned, water pack, solids and liquids (237)	237
	Dinner	20125	Pasta, whole-wheat, cooked , shells	52.5
		7933	Chicken breast, oven-roasted, fat-free, sliced (28.3)	84.9
		4053	Oil, olive, salad or cooking	13.5
		13217	Tomato sauce, canned, no salt added (245)	61.25
		11053	Beans, snap, green, cooked, boiled, drained, without salt (125)	125
		11305	Peas, green, cooked, boiled, drained, without salt (160)	80
		1032	Cheese, parmesan, grated (42.5)	10.625
	snacks	9302	Raspberries, raw (120)	30
		9040	Bananas, raw, sliced (150)	150
		1256	Yogurt, Greek, plain, nonfat (170)	170
		1085	Milk, nonfat, fluid, with added vitamin A and vitamin D (fat free or skim) (245)	122.5
6	Breakfast	1256	Yogurt, Greek, plain, nonfat (170)	170
		8037	Cereals ready-to-eat, granola, homemade (122) Made with oats, raisins, sunflower seeds, almonds, wheat germ, honey, coconut and canola oil.	61
		9302	Raspberries, raw (120)	60
		18061	Bread, rye, toasted, regular slice (24)	48
		9040	Bananas, raw, sliced (150)	150
	Lunch	16015	Beans, black, mature seeds, cooked, boiled, without salt (43)	43

continued on next page

continued from previous page

		20125	Pasta, whole-wheat, cooked , penne (48.5)	58.4
		11458	Spinach, cooked, boiled, drained, without salt (180)	45
		11283	Onions, cooked, boiled, drained, without salt (210)	52.5
		11168	Corn, sweet, yellow, cooked, boiled, drained, without salt, cut (149)	74.5
		11125	Carrots, cooked, boiled, drained, without salt (156)	78
	Dinner	13440	Beef, loin, tenderloin steak, boneless, separable lean and fat, trimmed to 0" fat, choice, cooked, grilled	113.2
		11091	Broccoli, cooked, boiled, drained, without salt, chopped (156)	78
		11371	Potatoes, mashed, home-prepared, whole milk and margarine added- (Recipe: Potatoes, 80% ; whole milk, 14% ; margarine, 5% ; and salt, 1% .) (210)	105
		11283	Onions, cooked, boiled, drained, without salt (210)	52.5
		11261	Mushrooms, white, cooked, boiled, drained, without salt (156)	39
		9286	Pomegranates, raw (174)	87
		1243	Fat free ice cream, no sugar added, flavors other than chocolate (204)	204
	Snacks	18216	Crackers, crispbread, rye, 2 crispbread (20)	30
		16158	Hummus, commercial	15
		1085	Milk, nonfat, fluid, with added vitamin A and vitamin D (fat free or skim) (245)	245
		18076	Bread, whole-wheat, commercially prepared, toasted, 1 slice, (25)	25
		4631	Margarine-like, vegetable oil spread, fat-free, tub (14.6) (0.44)	14.6
7	Breakfast	8037	Cereals ready-to-eat, granola, homemade (122) Made with oats, raisins, sunflower seeds, almonds, wheat germ, honey, coconut and canola oil.	61
		1085	Milk, nonfat, fluid, with added vitamin A and vitamin D (fat free or skim) (245)	122.5

continued on next page

continued from previous page

		9302	Raspberries, raw (120)	60
		9050	Blueberries, raw (148)	74
		18036	Bread, multi-grain, toasted (includes whole-grain), 1 slice, (24)	48
	Lunch	5168	Turkey, whole, meat only, cooked, roasted (28.3)	70.75
		20029	Couscous, cooked (78.5)	39.25
		11547	Tomato products, canned, puree, without salt added (250)	62.5
		11251	Lettuce, cos or romaine, raw, shredded (94)	23.5
		11821	Peppers, sweet, red, raw, chopped (149)	37.25
		11206	Cucumber, peeled, raw,sliced (119)	29.75
		4635	Salad dressing, thousand island dressing, fat-free	16
		1085	Milk, nonfat, fluid, with added vitamin A and vitamin D (fat free or skim) (245)	245
		9003	Apple, raw, with skin, 1 small small (2-3/4" diameter) (149)	149
	Dinner	23478	Beef, ground, 97% lean meat / 3% fat, patty, cooked, broiled (28.3)	84.9
		11805	Onions, cooked, boiled, drained, with salt (210)	52.5
		11125	Carrots, cooked, boiled, drained, without salt (156)	78
		11821	Peppers, sweet, red, raw, chopped (149)	37.25
		11305	Peas, green, cooked, boiled, drained, without salt (160)	80
		11357	Potatoes, white, flesh and skin, baked, medium (2-1/4" to 3-1/4" diameter) (173)	129.75
		9040	Bananas, raw, 1 large (8" to 8-7/8" long) (136)	136
	Snacks	18267	English muffins, whole-wheat, 1/2 english muffin toasted (30.5)	61
		4631	Margarine-like, vegetable oil spread, fat-free, tub (14.6) (0.44)	14.6
		16229	Soymilk (All flavors), lowfat, with added calcium, vitamins A and D (243)	243

continued on next page

continued from previous page

		18429	Crackers, whole-wheat, low salt, 5 crackers (20)	10
		1168	Cheese, low fat, cheddar or colby (42.5)	21.25
		19806	Snacks, popcorn, air-popped (Unsalted) (24)	24
8	Breakfast	8123	Cereals, oats, instant, fortified, plain, prepared with water (boiling water added or microwaved) (117)	175.5
		1085	Milk, nonfat, fluid, with added vitamin A and vitamin D (fat free or skim) (245)	122.5
		9302	Raspberries, raw (120)	30
		9050	Blueberries, raw (148)	37
		12037	Seeds, sunflower seed kernels, dry roasted, without salt (14.2)	14.2
		16398	Peanut butter, smooth style, without salt (16)	8
		18076	Bread, whole-wheat, commercially prepared, toasted, 1 slice, (25)	50
		4631	Margarine-like, vegetable oil spread, fat-free, tub (14.6) (0.44)	14.6
	Lunch	18042	Bread, pita, whole-wheat,1 small (4" diameter) (28)	56
		11252	Lettuce, iceberg (includes crisphead types), raw, chopped 1/2" pieces, loosely packed (114)	28.5
		5168	Turkey, whole, meat only, cooked, roasted (28.3)	70.75
		11821	Peppers, sweet, red, raw, chopped (149)	37.25
		1168	Cheese, low fat, cheddar or colby, shredded (37.7)	18.85
		43355	Mayonnaise, low sodium, low calorie or diet	14
		11529	Tomatoes, red, ripe, raw, year round average, cherry tomatoes (149)	37.25
		9096	Fruit cocktail, (peach and pineapple and pear and grape and cherry), canned, water pack, solids and liquids (237)	237
	Dinner	7933	Chicken breast, oven-roasted, fat-free, sliced (28.3)	42.45
		20125	Pasta, whole-wheat, cooked , shells (52.5)	26.25
		4053	Oil, olive, salad or cooking	13.5

continued on next page

continued from previous page

		11283	Onions, cooked, boiled, drained, without salt (210)	52.5
		11125	Carrots, cooked, boiled, drained, without salt (156)	39
		11144	Celery, cooked, boiled, drained, without salt, diced (150)	37.5
		11305	Peas, green, cooked, boiled, drained, without salt (160)	80
		11215	Garlic, raw clove	3
		14555	Water, bottled, generic,	474
		9003	Apple, raw, with skin, 1 small small (2-3/4" diameter) (149)	149
		11517	Sweet potato, frozen, cooked, baked, without salt, cubes (176)	132
	Snacks	9316	Strawberries, raw, 8 large (1-3/8" diameter), (144)	72
		1256	Yogurt, Greek, plain, nonfat (170)	170
		1085	Milk, nonfat, fluid, with added vitamin A and vitamin D (fat free or skim) (245)	367.5
		9040	Bananas, raw, 1 large (8" to 8-7/8" long) (136)	136
9	Breakfast	1256	Yogurt, Greek, plain, nonfat (170)	85
		9318	Strawberries, frozen, unsweetened, thawed (221)	110.5
		9054	Blueberries, frozen, unsweetened, unthawed (155)	38.75
		12037	Seeds, sunflower seed kernels, dry roasted, without salt (14.2)	7.1
		8122	Cereals, oats, instant, fortified, plain, dry, 1 packet (28)	28
		18061	Bread, rye, toasted, regular slice (24)	48
		4631	Margarine-like, vegetable oil spread, fat-free, tub (14.6) (0.44)	14.6
	Lunch	17012	Lamb, domestic, leg, whole (shank and sirloin), separable lean and fat, trimmed to 1/4" fat, choice, cooked, roasted	84.9
		20137	Quinoa, cooked	185
		16043	Beans, pinto, mature seeds, cooked, boiled, without salt (171)	42.75
		11821	Peppers, sweet, red, raw,1 Large, approx 3-3/4" long, 3" diameter (164)	82

continued on next page

continued from previous page

		11529	Tomatoes, red, ripe, raw, year round average, cherry tomatoes (149)	37.25
		11234	Kale, cooked, boiled, drained, without salt (130)	32.5
		11283	Onions, cooked, boiled, drained, without salt (210)	52.5
		1019	Cheese, feta, ctrumbled	37.5
		4053	Oil, olive, salad or cooking	13.5
	Dinner	20029	Couscous, cooked	157
		15116	Fish, trout, rainbow, wild, cooked, dry heat	56.6
		11099	Brussels sprouts, cooked, boiled, drained, without salt	78
		11367	Potatoes, boiled, cooked without skin, flesh, without salt (156)	39
		16057	Chickpeas (garbanzo beans, bengal gram), mature seeds, cooked, boiled, without salt	41
		4053	Oil, olive, salad or cooking	6.25
		1243	Fat free ice cream, no sugar added, flavors other than chocolate	204
		9236	Peaches, yellow, raw, Slices	77
	Snacks	9206	Orange juice, raw (248)	186
		1085	Milk, nonfat, fluid, with added vitamin A and vitamin D (fat free or skim) (245)	306.25
10	Breakfast	1256	Yogurt, Greek, plain, nonfat	170
		8037	Cereals ready-to-eat, granola, homemade (122)	61
		9050	Blueberries, raw (148)	74
		12037	Seeds, sunflower seed kernels, dry roasted, without salt (14.2)	7.1
		18061	Bread, rye, toasted, regular slice (24)	48
		4631	Margarine-like, vegetable oil spread, fat-free, tub (14.6) (0.44)	14.6
	Lunch	1130	Egg, whole, cooked, omelet (61)	61

continued on next page

continued from previous page

		11357	Potatoes, white, flesh and skin, baked, medium (2-1/4" to 3-1/4" diameter) (173)	173
		11283	Onions, cooked, boiled, drained, without salt (210)	52.5
		4053	Oil, olive, salad or cooking	13.5
		11529	Tomatoes, red, ripe, raw, year round average, cherry tomatoes (149)	37.25
		11261	Mushrooms, white, cooked, boiled, drained, without salt (156)	39
		11457	Spinach,raw (60)	15
		1168	Cheese, low fat, cheddar or colby, shredded (37.7)	18.85
		9096	Fruit cocktail, (peach and pineapple and pear and grape and cherry), canned, water pack, solids and liquids (237)	237
	Dinner	7933	Chicken breast, oven-roasted, fat-free, sliced	84.9
		20137	Quinoa, cooked	185
		11053	Beans, snap, green, cooked, boiled, drained, without salt	62.5
		11206	Cucumber, peeled, raw,sliced	29.75
		11032	Lima beans, immature seeds, cooked, boiled, drained, without salt	85
		11885	Tomatoes, red, ripe, canned, packed in tomato juice, no salt added (240)	120
		1220	Yogurt, vanilla, low fat, 11 grams protein per 8 ounce, fortified with vitamin D (245)	122.5
		9302	Raspberries, raw (120)	60
	Snacks	18216	Crackers, crispbread, rye, 2 crispbread (20)	30
		16158	Hummus, commercial (2 tbls) (30)	30
		1085	Milk, nonfat, fluid, with added vitamin A and vitamin D (fat free or skim) (245)	245
		9003	Apple, raw, with skin, 1 small small (2-3/4" diameter) (149)	149
11	Breakfsast	43449	Beans, baked, canned, no salt added (253)	126.5

continued on next page

continued from previous page

		18076	Bread, whole-wheat, commercially prepared, toasted	50
		9206	Orange juice, raw (248)	248
	Lunch	4053	Oil, olive, salad or cooking	6.25
		15047	Fish, mackerel, Atlantic, cooked, dry heat, (28.3)	84.9
		20037	Rice, brown, long-grain, cooked	151.5
		11510	Sweet potato, cooked, boiled, without skin, mashed	164
		12063	Nuts, almonds, dry roasted, without salt added (11 whole kernels) (14.2)	7.1
		9200	Oranges, raw, all commercial varieties, 1 large (3-1/16" diameter) (184)	184
		1256	Yogurt, Greek, plain, nonfat (170)	170
	Dinner	23478	Beef, ground, 97% lean meat / 3% fat, patty, cooked, broiled (28.3)	56.6
		13217	Tomato sauce, canned, no salt added (245)	61.25
		20125	Pasta, whole-wheat, cooked , shells (52.5)	52.5
		11091	Broccoli, cooked, boiled, drained, without salt, chopped (156)	39
		16028	Beans, kidney, all types, mature seeds, cooked, boiled, without salt (177)	88.5
		1085	Milk, nonfat, fluid, with added vitamin A and vitamin D (fat free or skim) (245)	245
		11168	Corn, sweet, yellow, cooked, boiled, drained, without salt, cut (149)	74.5
	Snacks	18216	Crackers, crispbread, rye, 2 crispbread (20)	30
		1168	Cheese, low fat, cheddar or colby, shredded (37.7)	18.85
		16229	Soymilk (All flavors), lowfat, with added calcium, vitamins A and D (243)	121.5
12	Breakfast	1130	Egg, whole, cooked, omelet (61)	61
		11921	Peppers, sweet, red, sauteed (106)	26.5

continued on next page

continued from previous page

		11529	Tomatoes, red, ripe, raw, year round average, cherry tomatoes (149)	74.5
		11457	Spinach,raw	15
		1168	Cheese, low fat, cheddar or colby, shredded (37.7)	37.7
		4053	Oil, olive, salad or cooking	13.5
		18061	Bread, rye, toasted, regular slice (24)	24
		4631	Margarine-like, vegetable oil spread, fat-free, tub (14.6) (0.44)	14.6
		1085	Milk, nonfat, fluid, with added vitamin A and vitamin D (fat free or skim) (245)	245
		9206	Orange juice, raw (248)	248
	Lunch	11252	Lettuce, iceberg (includes crisphead types), raw, chopped 1/2" pieces, loosely packed (114)	57
		11283	Onions, cooked, boiled, drained, without salt (210)	52.5
		17012	Lamb, domestic, leg, whole (shank and sirloin), separable lean and fat, trimmed to 1/4" fat, choice, cooked, roasted	56.6
		11206	Cucumber, peeled, raw,sliced (119)	29.75
		11124	Carrots, raw, grated (110)	27.5
		4635	Salad dressing, thousand island dressing, fat-free	16
		20029	Couscous, cooked (78.5)	157
		1085	Milk, nonfat, fluid, with added vitamin A and vitamin D (fat free or skim) (245)	245
		9040	Bananas, raw, 1 large (8" to 8-7/8" long) (136)	136
	Dinner	15151	Crustaceans, shrimp, mixed species, cooked, moist heat (may have been previously frozen)	84.9
		20125	Pasta, whole-wheat, cooked , spaghetti not packed (58.5)	87.75
		11458	Spinach, cooked, boiled, drained, without salt (180)	45
		4053	Oil, olive, salad or cooking	6.75
		18061	Bread, rye, toasted, regular slice (24)	24

continued on next page

continued from previous page

		9252	Pears, raw,1 medium (178)	178
	Snacks	19806	Snacks, popcorn, air-popped (Unsalted) (24)	12
		1085	Milk, nonfat, fluid, with added vitamin A and vitamin D (fat free or skim) (245)	245
		9132	Grapes,32 red or green (European type, such as Thompson seedless), raw (156.8)	78.4
13	Breakfast	8123	Cereals, oats, instant, fortified, plain, prepared with water (boiling water added or microwaved) (117)	175.5
		1085	Milk, nonfat, fluid, with added vitamin A and vitamin D (fat free or skim) (245)	245
		9302	Raspberries, raw (120)	30
		9050	Blueberries, raw (148)	37
		12037	Seeds, sunflower seed kernels, dry roasted, without salt (14.2)	7.1
	Lunch	5168	Turkey, whole, meat only, cooked, roasted (28.3)	56.6
		11921	Peppers, sweet, red, sauteed (106)	26.5
		11529	Tomatoes, red, ripe, raw, year round average, cherry tomatoes (149)	37.25
		11457	Spinach,raw	15
		11333	Peppers, sweet, green, raw,1 large (2-1/4 per lb, approx 3-3/4" long, 3" diameter) (164)	41
		4053	Oil, olive, salad or cooking	6.75
		20125	Pasta, whole-wheat, cooked , shells (52.5)	52.5
	dinner	23478	Beef, ground, 97% lean meat / 3% fat, patty, cooked, broiled (28.3)	84.9
		11091	Broccoli, cooked, boiled, drained, without salt, chopped (156)	39
		11371	Potatoes, mashed, home-prepared, whole milk and margarine added- (Recipe: Potatoes, 80% ; whole milk, 14% ; margarine, 5% ; and salt, 1% .) (210)	157.5
		11283	Onions, cooked, boiled, drained, without salt (210)	52.5

continued on next page

continued from previous page

		11261	Mushrooms, white, cooked, boiled, drained, without salt (156)	117
		4053	Oil, olive, salad or cooking	6.75
		9286	Pomegranates, raw (174)	87
		1243	Fat free ice cream, no sugar added, flavors other than chocolate (204)	204
	Snacks	18267	English muffins, whole-wheat, 1/2 english muffin toasted (30.5)	61
		18216	Crackers, crispbread, rye, 2 crispbread (20)	30
		1256	Yogurt, Greek, plain, nonfat (170)	170
		9316	Strawberries, raw, 8 large (1-3/8" diameter), (144)	144
		9003	Apple, raw, with skin, 1 small small (2-3/4" diameter) (149)	149
		16229	Soymilk (All flavors), lowfat, with added calcium, vitamins A and D (243)	243
14	Breakfast	9037	Avocados, raw, all commercial varieties, sliced (146)	36.5
		18061	Bread, rye, toasted, regular slice (24)	48
		1131	Egg, whole, cooked, poached (1 large) (50)	50
		1131	Egg, whole, cooked, poached (1 large) (50)	50
		9206	Orange juice, raw (248)	248
	Lunch	11357	Potatoes, white, flesh and skin, baked, medium (2-1/4" to 3-1/4" diameter) (173)	173
		7933	Chicken breast, oven-roasted, fat-free, sliced (28.3)	56.6
		11168	Corn, sweet, yellow, cooked, boiled, drained, without salt,1 ear small (5-1/2" to 6-1/2" long)	74.5
		11251	Lettuce, cos or romaine, raw	23.5
		11529	Tomatoes, red, ripe, raw, year round average, cherry tomatoes	74.5
		16229	Soymilk (All flavors), lowfat, with added calcium, vitamins A and D	243

continued on next page

continued from previous page

	Dinner	23478	Beef, ground, 97% lean meat / 3% fat, patty, cooked, broiled (28.3)	56.6
		4053	Oil, olive, salad or cooking	13.5
		11283	Onions, cooked, boiled, drained, without salt (210)	52.5
		11547	Tomato products, canned, puree, without salt added (250)	62.5
		20125	Pasta, whole-wheat, cooked , spaghetti not packed (58.5)	87.75
		11125	Carrots, cooked, boiled, drained, without salt (156)	39
		1168	Cheese, low fat, cheddar or colby, shredded (37.7)	18.85
		9252	Pears, raw,1 medium (178)	178
		1256	Yogurt, Greek, plain, nonfat (170)	170
	Snacks	16158	Hummus, commercial (2 tbls) (30)	15
		18235	Crackers, whole-wheat, 5 crackers (23)	11.5
		1085	Milk, nonfat, fluid, with added vitamin A and vitamin D (fat free or skim) (245)	245
		18076	Bread, whole-wheat, commercially prepared, toasted	50
		1168	Cheese, low fat, cheddar or colby, shredded (37.7)	18.85

Vegetarian-style menu plan

Table 4: 8

Day	Meal	Usda	Item	Grams
1	Breakfast	18061	Bread, rye, toasted, regular slice (24)	48
		1129	Egg, whole, cooked, hard-boiled, 1 large egg (50)	50
		1168	Cheese, low fat, cheddar or colby, shredded (37.7)	18.85
		1085	Milk, nonfat, fluid, with added vitamin A and vitamin D (fat free or skim) (245)	122.5
		9200	Oranges, raw, all commercial varieties, 2 small (2-3/8" diameter)(96)	48

continued on next page

continued from previous page

	Lunch	18076	Bread, whole-wheat, commercially prepared, toasted, 1 slice, (25)	50
		1168	Cheese, low fat, cheddar or colby, shredded (37.7)	37.7
		11529	Tomatoes, red, ripe, raw, year round average, 1 large whole (3" diameter) (123)	61.5
		11179	Corn, sweet, yellow, frozen, kernels cut off cob, boiled, drained, without salt (165)	82.5
		11457	Spinach,raw	15
		1085	Milk, nonfat, fluid, with added vitamin A and vitamin D (fat free or skim) (245)	245
		9003	Apples, raw, with skin, quatered or chopped (red delicious, golden delicious, gala, granny smith, and fuji varieties) (125)	125
	Dinner	20137	Quinoa, cooked	185
		16043	Beans, pinto, mature seeds, cooked, boiled, without salt (171)	42.75
		11821	Peppers, sweet, red, raw,1 Large, approx 3-3/4" long, 3" diameter (164)	74.5
		11529	Tomatoes, red, ripe, raw, year round average, cherry tomatoes (149)	37.25
		11234	Kale, cooked, boiled, drained, without salt (130)	32.5
		11805	Onions, cooked, boiled, drained, with salt (210)	52.5
		1019	Cheese, feta, ctrumbled	37.5
		4053	Oil, olive, salad or cooking	13.5
		12016	Seeds, pumpkin and squash seed kernels, roasted, without salt (14.2)	14.2
		9200	Oranges, raw, all commercial varieties, 2 small (2-3/8" diameter)(96)	48
	Snacks	18216	Crackers, crispbread, rye, 2 crispbread (20)	10
		16158	Hummus, commercial (2 tbls) (30)	15
		12155	Nuts, walnuts, english, 7 halves (14.2)	14.2
		16229	Soymilk (All flavors), lowfat, with added calcium, vitamins A and D	182.25
	Water	14555	Water, bottled, generic	2000
2	Breakfast	1256	Yogurt, Greek, plain, nonfat (170)	170
		8122	Cereals, oats, instant, fortified, plain, dry, 1 packet (28)	28
		9050	Blueberries, raw (148)	74

continued on next page

continued from previous page

		12037	Seeds, sunflower seed kernels, dry roasted, without salt	7.1
		18076	Bread, whole-wheat, commercially prepared, toasted, 1 slice, (25)	50
		4697	Margarine-like, vegetable oil spread, 60% fat, stick/tub/bottle, without salt, with added vitamin D (14g) 1 tbls	7
		16229	Soymilk (All flavors), lowfat, with added calcium, vitamins A and D	121.5
	Lunch	20137	Quinoa, cooked (92.5)	92.5
		16070	Lentils, mature seeds, cooked, boiled, without salt (49.59)	49.5
		16017	Beans, black turtle, mature seeds, cooked, boiled, without salt (185)	46.25
		11251	Lettuce, cos or romaine, raw, shredded (94)	47
		11206	Cucumber, peeled, raw,sliced (119)	29.75
		9195	Olives, pickled, canned or bottled, green, 1 olive (2.7g)) (0.41g oil)	10.8
		4635	Salad dressing, thousand island dressing, fat-free	16
		9266	Pineapple, raw, all varieties, chunks (165)	82.5
	Dinner	1130	Egg, whole, cooked, omelet (61)	61
		11357	Potatoes, white, flesh and skin, baked, medium (2-1/4" to 3-1/4" diameter) (173)	86.5
		11283	Onions, cooked, boiled, drained, without salt (210)	52.5
		4053	Oil, olive, salad or cooking	13.5
		11529	Tomatoes, red, ripe, raw, year round average, cherry tomatoes (149)	37.25
		11261	Mushrooms, white, cooked, boiled, drained, without salt (156)	39
		11457	Spinach,raw (60)	15
		1168	Cheese, low fat, cheddar or colby, shredded (37.7)	18.85
		9097	Fruit cocktail, (peach and pineapple and pear and grape and cherry), canned, juice pack, solids and liquids	237
	Snacks	1220	Yogurt, vanilla, low fat, 11 grams protein per 8 ounce, fortified with vitamin D (245)	245
		16158	Hummus, commercial (2 tbls) (30)	30
		18429	Crackers, whole-wheat, low salt, 5 crackers (20)	20
		18036	Bread, multi-grain, toasted (includes whole-grain), 1 slice, (24)	24

continued on next page

continued from previous page

		16398	Peanut butter, smooth style, without salt (16)	8
3	Breakfast	1130	Egg, whole, cooked, omelet (61)	61
		11478	Squash, summer, zucchini, includes skin, cooked, boiled, drained, without salt, sliced (180)	45
		11282	Onions, raw,chopped (160)	40
		11261	Mushrooms, white, cooked, boiled, drained, without salt (156)	39
		11234	Kale, cooked, boiled, drained, without salt (130)	32.5
		11821	Peppers, sweet, red, raw,1 Large, approx 3-3/4" long, 3" diameter (164)	37.25
		11547	Tomato products, canned, puree, without salt added (250)	62.5
		1168	Cheese, low fat, cheddar or colby, shredded (37.7)	18.85
		18036	Bread, multi-grain, toasted (includes whole-grain), 1 slice, (24)	24
		4697	Margarine-like, vegetable oil spread, 60% fat, stick/tub/bottle, without salt, with added vitamin D (14g) 1 tbls	7
		9206	Orange juice, raw (248)	248
	Lunch	28318	Bread, french or vienna, whole wheat,1 slice	96
		6192	Split pea soup, canned, reduced sodium, prepared with water or ready-to serve	126.5
		9286	Pomegranates, raw	43.5
		9040	Bananas, raw, sliced (150)	75
		1243	Fat free ice cream, no sugar added, flavors other than chocolate	102
	Dinner	20125	Pasta, whole-wheat, cooked , spaghetti not packed (58.5)	117
		11261	Mushrooms, white, cooked, boiled, drained, without salt (156)	39
		11179	Corn, sweet, yellow, frozen, kernels cut off cob, boiled, drained, without salt (165)	82.5
		11885	Tomatoes, red, ripe, canned, packed in tomato juice, no salt added (240)	60
		11125	Carrots, cooked, boiled, drained, without salt (156)	78
		1032	Cheese, parmesan, grated	21.25
		1220	Yogurt, vanilla, low fat, 11 grams protein per 8 ounce, fortified with vitamin D (245)	245
		9316	Strawberries, raw, sliced (166)	41.5

continued on next page

continued from previous page

	Snacks	18216	Crackers, crispbread, rye, 2 crispbread (20)	20
		16158	Hummus, commercial (2 tbls) (30)	15
		1085	Milk, nonfat, fluid, with added vitamin A and vitamin D (fat free or skim) (245)	245
		12155	Nuts, walnuts, english, 7 halves (14.2)	7.1
4	Breakfast	8123	Cereals, oats, instant, fortified, plain, prepared with water (boiling water added or microwaved) (117)	117
		12037	Seeds, sunflower seed kernels, dry roasted, without salt (14.2)	7.1
		1256	Yogurt, Greek, plain, nonfat (170)	170
		9302	Raspberries, raw (120)	60
		18061	Bread, rye, toasted, regular slice (24)	48
		9206	Orange juice, raw (248)	248
	Lunch	16126	Tofu, firm, prepared with calcium sulfate and magnesium chloride (nigari) (63)	63
		11099	Brussels sprouts, cooked, boiled, drained, without salt (156)	78
		20112	Noodles, egg, spinach, enriched, cooked (80)	120
		4058	Oil, sesame, salad or cooking	6.8
		16424	Soy sauce made from soy and wheat (shoyu), low sodium (1 tbls) 14.2g	14.2
		11091	Broccoli, cooked, boiled, drained, without salt, chopped (156)	39
		9326	Watermelon, raw, diced (152)	76
	Dinner	20029	Couscous, cooked	157.6
		4053	Oil, olive, salad or cooking	13.5
		11032	Lima beans, immature seeds, cooked, boiled, drained, without salt	85
		16043	Beans, pinto, mature seeds, cooked, boiled, without salt (171)	85.5
		11529	Tomatoes, red, ripe, raw, year round average, 1 large whole (3" diameter) (123)	61.5
		11333	Peppers, sweet, green, raw,chopped	74.5
		9195	Olives, pickled, canned or bottled, green, 1 olive	10.8
		9266	Pineapple, raw, all varieties, chunks (165)	82.5

continued on next page

continued from previous page

	Snacks	1085	Milk, nonfat, fluid, with added vitamin A and vitamin D (fat free or skim) (245)	490
		18216	Crackers, crispbread, rye, 2 crispbread (20)	20
day 5	Breakfast	1256	Yogurt, Greek, plain, nonfat (170)	170
		9318	Strawberries, frozen, unsweetened, thawed (221)	110.5
		9054	Blueberries, frozen, unsweetened, unthawed (155)	38.75
		12037	Seeds, sunflower seed kernels, dry roasted, without salt (14.2)	7.1
		8122	Cereals, oats, instant, fortified, plain, dry, 1 packet (28)	28
		18061	Bread, rye, toasted, regular slice (24)	48
		9206	Orange juice, raw (248)	248
	Lunch	18076	Bread, whole-wheat, commercially prepared, toasted, 1 slice, (25)	50
		4697	Margarine-like, vegetable oil spread, 60% fat, stick/tub/bottle, without salt, with added vitamin D (14g) 1 tbls	7
		1168	Cheese, low fat, cheddar or colby, shredded (37.7)	37.7
		11529	Tomatoes, red, ripe, raw, year round average, 1 large whole (3" diameter) (123)	61.5
		11179	Corn, sweet, yellow, frozen, kernels cut off cob, boiled, drained, without salt (165)	82.5
	Dinner	16070	Lentils, mature seeds, cooked, boiled, without salt (49.59)	49.5
		4053	Oil, olive, salad or cooking	13.5
		11283	Onions, cooked, boiled, drained, without salt (210)	52.5
		11478	Squash, summer, zucchini, includes skin, cooked, boiled, drained, without salt, sliced (180)	45
		11821	Peppers, sweet, red, raw,1 Large, approx 3-3/4" long, 3" diameter (164)	37.25
		16057	Chickpeas (garbanzo beans, bengal gram), mature seeds, cooked, boiled, without salt (164)	41
		11179	Corn, sweet, yellow, frozen, kernels cut off cob, boiled, drained, without salt (165)	82.5
		11032	Lima beans, immature seeds, cooked, boiled, drained, without salt	85
		11885	Tomatoes, red, ripe, canned, packed in tomato juice, no salt added (240)	120
		9097	Fruit cocktail, (peach and pineapple and pear and grape and cherry), canned, juice pack, solids and liquids (237)	118.5

continued on next page

continued from previous page

	Snacks	12155	Nuts, walnuts, english, 7 halves (14.2)	14.2
		19806	Snacks, popcorn, air-popped (Unsalted) (24)	36
		1220	Yogurt, vanilla, low fat, 11 grams protein per 8 ounce, fortified with vitamin D (245)	245
		9050	Blueberries, raw (148)	74
Day 6	Breakfast	1256	Yogurt, Greek, plain, nonfat (170)	170
		8037	Cereals ready-to-eat, granola, homemade (122) Made with oats, raisins, sunflower seeds, almonds, wheat germ, honey, coconut and canola oil.	61
		9302	Raspberries, raw (120)	60
		18061	Bread, rye, toasted, regular slice (24)	24
		9040	Bananas, raw, sliced (150)	150
		16229	Soymilk (All flavors), lowfat, with added calcium, vitamins A and D (243)	243
	Lunch	28318	Bread, french or vienna, whole wheat,1 slice	96
		6192	Split pea soup, canned, reduced sodium, prepared with water or ready-to serve	126.5
		9266	Pineapple, raw, all varieties, chunks (165)	82.5
	dinner	16126	Tofu, firm, prepared with calcium sulfate and magnesium chloride (nigari) (63)	63
		11091	Broccoli, cooked, boiled, drained, without salt, chopped (156)	39
		11125	Carrots, cooked, boiled, drained, without salt (156)	78
		11053	Beans, snap, green, cooked, boiled, drained, without salt (125)	62.5
		11305	Peas, green, cooked, boiled, drained, without salt (160)	40
		16328	Beans, kidney, all types, mature seeds, cooked, boiled, with salt (177)	44.25
		20112	Noodles, egg, spinach, enriched, cooked (80)	120
		9037	Avocados, raw, all commercial varieties, cubes (150)	75
		4058	Oil, sesame, salad or cooking	6.8
		16424	Soy sauce made from soy and wheat (shoyu), low sodium (1 tbls) 14.2g	14.2
	Snacks	18216	Crackers, crispbread, rye, 2 crispbread (20)	30
		11529	Tomatoes, red, ripe, raw, year round average, cherry tomatoes (149)	37.25

continued on next page

continued from previous page

		12195	Nuts, almond butter, plain, without salt added (16)	24
		12155	Nuts, walnuts, english, 7 halves (14.2)	7.1
		1085	Milk, nonfat, fluid, with added vitamin A and vitamin D (fat free or skim) (245)	245
day7	Breakfast	8123	Cereals, oats, instant, fortified, plain, prepared with water (boiling water added or microwaved) (117)	0
		1085	Milk, nonfat, fluid, with added vitamin A and vitamin D (fat free or skim) (245)	0
		18076	Bread, whole-wheat, commercially prepared, toasted, 1 slice, (25)	2.373
		4697	Margarine-like, vegetable oil spread, 60% fat, stick/tub/bottle, without salt, with added vitamin D (14g) 1 tbls	0
		1256	Yogurt, Greek, plain, nonfat (170)	0
		9050	Blueberries, raw (148)	0
		12037	Seeds, sunflower seed kernels, dry roasted, without salt	0
		9040	Bananas, raw, 1 large (8" to 8-7/8" long) (136)	0
	Lunch	20029	Couscous, cooked	0
		16070	Lentils, mature seeds, cooked, boiled, without salt (49.5)	0
		16015	Beans, black, mature seeds, cooked, boiled, without salt (172)	0
		16057	Chickpeas (garbanzo beans, bengal gram), mature seeds, cooked, boiled, without salt	0
		11305	Peas, green, cooked, boiled, drained, without salt (160)	0
		11529	Tomatoes, red, ripe, raw, year round average, cherry tomatoes (149)	0
		9132	Grapes,32 red or green (European type, such as Thompson seedless), raw (156.8)	0
	Dinner	20037	Rice, brown, long-grain, cooked (101)	0
		11508	Sweet potato, cooked, baked in skin, flesh, without salt (200)	0
		11357	Potatoes, white, flesh and skin, baked, medium (2-1/4" to 3-1/4" diameter) (173)	0
		11305	Peas, green, cooked, boiled, drained, without salt (160)	0
		11529	Tomatoes, red, ripe, raw, year round average, cherry tomatoes (149)	0
		2015	Spices, curry powder 1 tbls (6.3g)	0
		1256	Yogurt, Greek, plain, nonfat (170)	0

continued on next page

continued from previous page

		1256	Yogurt, Greek, plain, nonfat (170)	0
		9286	Pomegranates, raw	0
		9132	Grapes,32 red or green (European type, such as Thompson seedless), raw (156.8)	0
	Snacks	1220	Yogurt, vanilla, low fat, 11 grams protein per 8 ounce, fortified with vitamin D (245)	16.6698
		12016	Seeds, pumpkin and squash seed kernels, roasted, without salt (14.2)	0
		9181	Melons, cantaloupe, raw, balls (177)	0
		12063	Nuts, almonds, dry roasted, without salt added (11 whole kernels) (14.2)	0
day8	Breakfast	9037	Avocados, raw, all commercial varieties, sliced	73
		18061	Bread, rye, toasted, regular slice	48
		1168	Cheese, low fat, cheddar or colby, shredded	18.85
		1131	Egg, whole, cooked, poached (1 large)	50
		1220	Yogurt, vanilla, low fat, 11 grams protein per 8 ounce, fortified with vitamin D	122.5
		9040	Bananas, raw, 1 large	136
		9316	Strawberries, raw, 8 large	122
		9206	Orange juice, raw	124.5
	Lunch	16057	Chickpeas (garbanzo beans, bengal gram), mature seeds, cooked, boiled, without salt	41
		11251	Lettuce, cos or romaine, raw, shredded	23.3
		11821	Peppers, sweet, red, raw,1 Large, approx 3-3/4" long, 3" diameter	37.25
		11529	Tomatoes, red, ripe, raw, year round average, cherry tomatoes	37.25
		11206	Cucumber, peeled, raw,sliced	29.75
		12016	Seeds, pumpkin and squash seed kernels, roasted, without salt	7.1
		4635	Salad dressing, thousand island dressing, fat-free	16
		28397	Bread, multi-grain (includes whole-grain),1 slice regular (26)	26
	Dinner	18042	Bread, pita, whole-wheat,1 small (4" diameter)	56
		11547	Tomato products, canned, puree, without salt added (250)	62.5
		1029	Cheese, mozzarella, low moisture, part-skim, sliced (42)	42

continued on next page

continued from previous page

		11821	Peppers, sweet, red, raw,1 Large, approx 3-3/4" long, 3" diameter (164)	37.25
		11457	Spinach,raw	15
		9195	Olives, pickled, canned or bottled, green, 1 olive (2.7g)) (0.41g oil)	10.8
		6192	Split pea soup, canned, reduced sodium, prepared with water or ready-to serve (126.5)	126.5
		1243	Fat free ice cream, no sugar added, flavors other than chocolate (204)	102
	Snacks	19806	Snacks, popcorn, air-popped (Unsalted) (24)	36
		12152	Nuts, pistachio nuts, dry roasted, without salt added, 24 kernels (14.2)	14.2
		12063	Nuts, almonds, dry roasted, without salt added (11 whole kernels) (14.2)	14.2
		16229	Soymilk (All flavors), lowfat, with added calcium, vitamins A and D (243)	121.5
day9	Breakfast	1256	Yogurt, Greek, plain, nonfat	170
		8037	Cereals ready-to-eat, granola, homemade (122)	61
		9050	Blueberries, raw (148)	74
		12037	Seeds, sunflower seed kernels, dry roasted, without salt (14.2)	7.1
		18061	Bread, rye, toasted, regular slice (24)	24
	Lunch	18449	Tortillas, ready-to-bake or -fry, corn, without added salt (approx 6" diameter) (26)	26
		9037	Avocados, raw, all commercial varieties, sliced (146)	36.5
		16015	Beans, black, mature seeds, cooked, boiled, without salt (172)	86
		11821	Peppers, sweet, red, raw,1 Large, approx 3-3/4" long, 3" diameter (164)	37.25
		11283	Onions, cooked, boiled, drained, without salt (210)	52.5
		1168	Cheese, low fat, cheddar or colby, shredded (37.7)	18.85
		6164	Sauce, salsa, ready-to-serve (18) 1 tbls	18
		11517	Sweet potato, frozen, cooked, baked, without salt, cubes (176)	44
		1085	Milk, nonfat, fluid, with added vitamin A and vitamin D (fat free or skim) (245)	245
		9266	Pineapple, raw, all varieties, chunks (165)	82.5
	Dinner	16126	Tofu, firm, prepared with calcium sulfate and magnesium chloride (nigari) (63)	63
		11099	Brussels sprouts, cooked, boiled, drained, without salt (156)	78

continued on next page

continued from previous page

		20112	Noodles, egg, spinach, enriched, cooked (80)	120
		4058	Oil, sesame, salad or cooking	6.8
		16424	Soy sauce made from soy and wheat (shoyu), low sodium (1 tbls) 14.2g	14.2
		11091	Broccoli, cooked, boiled, drained, without salt, chopped (156)	39
		11053	Beans, snap, green, cooked, boiled, drained, without salt (125)	31.25
		9326	Watermelon, raw, diced (152)	76
	Snacks	18429	Crackers, whole-wheat, low salt, 5 crackers (20)	40
		16398	Peanut butter, smooth style, without salt (16)	16
		9200	Oranges, raw, all commercial varieties, 2 small (2-3/8" diameter)(96)	96
		16229	Soymilk (All flavors), lowfat, with added calcium, vitamins A and D (243)	121.5
		9003	Apple, raw, with skin, 1 small small (2-3/4" diameter) (149)	149
		18216	Crackers, crispbread, rye, 2 crispbread (20)	10
day10	Breakfast	1130	Egg, whole, cooked, omelet (61)	61
		11478	Squash, summer, zucchini, includes skin, cooked, boiled, drained, without salt, sliced (180)	45
		11261	Mushrooms, white, cooked, boiled, drained, without salt (156)	39
		11234	Kale, cooked, boiled, drained, without salt (130)	32.5
		11821	Peppers, sweet, red, raw,1 Large, approx 3-3/4" long, 3" diameter (164)	37.25
		11547	Tomato products, canned, puree, without salt added (250)	62.5
		18036	Bread, multi-grain, toasted (includes whole-grain), 1 slice, (24)	48
		4631	Margarine-like, vegetable oil spread, fat-free, tub (14.6) (0.44)	14.6
		9206	Orange juice, raw (248)	248
	Lunch	16070	Lentils, mature seeds, cooked, boiled, without salt (49.59)	74.25
		11125	Carrots, cooked, boiled, drained, without salt (156)	78
		11885	Tomatoes, red, ripe, canned, packed in tomato juice, no salt added (240)	60
		4053	Oil, olive, salad or cooking	13.5
		11357	Potatoes, white, flesh and skin, baked, medium (2-1/4" to 3-1/4" diameter) (173)	86.5

continued on next page

continued from previous page

		11357	Potatoes, white, flesh and skin, baked, medium (2-1/4" to 3-1/4" diameter) (173)	86.5
		1180	Sour cream, fat free (12g) 1 tbls	12
		1085	Milk, nonfat, fluid, with added vitamin A and vitamin D (fat free or skim) (245)	245
		9326	Watermelon, raw, 12 balls (146.4)	73.2
	Dinner	16126	Tofu, firm, prepared with calcium sulfate and magnesium chloride (nigari) (63)	63
		20037	Rice, brown, long-grain, cooked (101)	151.5
		11125	Carrots, cooked, boiled, drained, without salt (156)	78
		11053	Beans, snap, green, cooked, boiled, drained, without salt (125)	62.5
		4058	Oil, sesame, salad or cooking	6.8
		28318	Bread, french or vienna, whole wheat,1 slice (48)	52
		9003	Apple, raw, with skin, 1 small small (2-3/4" diameter) (149)	149
		1243	Fat free ice cream, no sugar added, flavors other than chocolate	102
	Snacks	1256	Yogurt, Greek, plain, nonfat	85
		9326	Watermelon, raw, 12 balls (146.4)	146.4
		1085	Milk, nonfat, fluid, with added vitamin A and vitamin D (fat free or skim) (245)	245
		19806	Snacks, popcorn, air-popped (Unsalted) (24)	24
day11	Breakfast	1130	Egg, whole, cooked, omelet (61)	61
		11011	Asparagus, raw (134)	33.5
		11921	Peppers, sweet, red, sauteed (106)	26.4
		11529	Tomatoes, red, ripe, raw, year round average, cherry tomatoes (149)	37.25
		11457	Spinach,raw	15
		1168	Cheese, low fat, cheddar or colby, shredded (37.7)	37.7
		18036	Bread, multi-grain, toasted (includes whole-grain), 1 slice, (24)	48
		4631	Margarine-like, vegetable oil spread, fat-free, tub (14.6) (0.44)	14.6
		1085	Milk, nonfat, fluid, with added vitamin A and vitamin D (fat free or skim) (245)	245
	Lunch	18042	Bread, pita, whole-wheat,1 small (4" diameter) (28)	56

continued on next page

continued from previous page

		11206	Cucumber, peeled, raw,sliced (119)	29.75
		11283	Onions, cooked, boiled, drained, without salt (210)	52.5
		16138	Falafel, home-prepared, patty (approx 2-1/4" diameter) (17)	17
		4053	Oil, olive, salad or cooking	6.75
		9252	Pears, raw,1 medium	178
	Dinner	20029	Couscous, cooked	157.6
		11032	Lima beans, immature seeds, cooked, boiled, drained, without salt	42.5
		16043	Beans, pinto, mature seeds, cooked, boiled, without salt (171)	61.125
		11529	Tomatoes, red, ripe, raw, year round average, 1 large whole (3" diameter) (123)	61.5
		11333	Peppers, sweet, green, raw,chopped	74.5
		9195	Olives, pickled, canned or bottled, green, 1 olive	10.8
		4053	Oil, olive, salad or cooking	13.5
		28318	Bread, french or vienna, whole wheat,1 slice (48)	26
		9040	Bananas, raw, 1 large (8" to 8-7/8" long) (136)	136
	Snacks	18216	Crackers, crispbread, rye, 2 crispbread (20)	30
		16158	Hummus, commercial (2 tbls) (30)	30
		1085	Milk, nonfat, fluid, with added vitamin A and vitamin D (fat free or skim) (245)	245
		1220	Yogurt, vanilla, low fat, 11 grams protein per 8 ounce, fortified with vitamin D (245)	122.5
		9316	Strawberries, raw, halves (152)	152
day12	Breakfast	1256	Yogurt, Greek, plain, nonfat (170)	85
		8122	Cereals, oats, instant, fortified, plain, dry, 1 packet (28)	14
		9040	Bananas, raw, 1 large (8" to 8-7/8" long) (136)	136
		9316	Strawberries, raw, 8 large (1-3/8" diameter), (144)	122
		9206	Orange juice, raw (248)	124.5
		12037	Seeds, sunflower seed kernels, dry roasted, without salt (14.2)	14.2
		12016	Seeds, pumpkin and squash seed kernels, roasted, without salt (14.2)	7.1

continued on next page

continued from previous page

		18076	Bread, whole-wheat, commercially prepared, toasted, 1 slice, (25)	50
		16398	Peanut butter, smooth style, without salt (16)	16
	Lunch	11210	Eggplant, cooked, boiled, drained, without salt (99)	49.5
		11305	Peas, green, cooked, boiled, drained, without salt (160)	80
		11529	Tomatoes, red, ripe, raw, year round average, 1 large whole (3" diameter) (123)	61.5
		1029	Cheese, mozzarella, low moisture, part-skim, shredded (28.7)	28.7
		20037	Rice, brown, long-grain, cooked (101)	101
		16229	Soymilk (All flavors), lowfat, with added calcium, vitamins A and D (243)	243
		9097	Fruit cocktail, (peach and pineapple and pear and grape and cherry), canned, juice pack, solids and liquids (237)	121.5
	Dinner	16126	Tofu, firm, prepared with calcium sulfate and magnesium chloride (nigari) (63)	63
		20112	Noodles, egg, spinach, enriched, cooked (80)	120
		11091	Broccoli, cooked, boiled, drained, without salt, chopped (156)	39
		11125	Carrots, cooked, boiled, drained, without salt (156)	78
		11053	Beans, snap, green, cooked, boiled, drained, without salt (125)	62.5
		11305	Peas, green, cooked, boiled, drained, without salt (160)	40
		16028	Beans, kidney, all types, mature seeds, cooked, boiled, without salt (177)	44.25
		4058	Oil, sesame, salad or cooking	6.8
		16424	Soy sauce made from soy and wheat (shoyu), low sodium (1 tbls) 14.2g	14.2
	Snacks	16229	Soymilk (All flavors), lowfat, with added calcium, vitamins A and D (243)	121.5
		18061	Bread, rye, toasted, regular slice (24)	48
		28399	Cookies, animal crackers (includes arrowroot, tea biscuits), 7 biscuits	8.75
day13	Breakfast	8123	Cereals, oats, instant, fortified, plain, prepared with water (boiling water added or microwaved) (117)	117
		12037	Seeds, sunflower seed kernels, dry roasted, without salt (14.2)	7.1
		1256	Yogurt, Greek, plain, nonfat (170)	85
		9302	Raspberries, raw (120)	60

continued on next page

continued from previous page

		12195	Nuts, almond butter, plain, without salt added 1/2 tbls	8
		18061	Bread, rye, toasted, regular slice (24)	48
		9003	Apple, raw, with skin, 1 small small (2-3/4" diameter) (149)	149
	Lunch	20029	Couscous, cooked	138.75
		16070	Lentils, mature seeds, cooked, boiled, without salt (49.59)	49.5
		16015	Beans, black, mature seeds, cooked, boiled, without salt (172)	43
		16057	Chickpeas (garbanzo beans, bengal gram), mature seeds, cooked, boiled, without salt	82
		11305	Peas, green, cooked, boiled, drained, without salt (160)	80
		11821	Peppers, sweet, red, raw, chopped (149)	37.25
		1019	Cheese, feta, crumbled (150)	37.5
		16229	Soymilk (All flavors), lowfat, with added calcium, vitamins A and D (243)	121.5
	Dinner	11357	Potatoes, white, flesh and skin, baked, medium (2-1/4" to 3-1/4" diameter) (173)	173
		11305	Peas, green, cooked, boiled, drained, without salt (160)	160
		11529	Tomatoes, red, ripe, raw, year round average, cherry tomatoes (149)	37.25
		11179	Corn, sweet, yellow, frozen, kernels cut off cob, boiled, drained, without salt (165)	82.5
		20125	Pasta, whole-wheat, cooked , shells (52.5)	52.5
		11547	Tomato products, canned, puree, without salt added (250)	62.5
		1032	Cheese, parmesan, grated (42.5)	21.25
		9266	Pineapple, raw, all varieties, chunks (165)	82.5
	Snacks	18216	Crackers, crispbread, rye, 2 crispbread (20)	20
		16158	Hummus, commercial (2 tbls) (30)	30
		1085	Milk, nonfat, fluid, with added vitamin A and vitamin D (fat free or skim) (245)	245
day14	Breakfast	43449	Beans, baked, canned, no salt added (253)	126.5
		18076	Bread, whole-wheat, commercially prepared, toasted	50
		4631	Margarine-like, vegetable oil spread, fat-free, tub (14.6) (0.44)	7.3
		1085	Milk, nonfat, fluid, with added vitamin A and vitamin D (fat free or skim) (245)	245

continued on next page

continued from previous page

		9040	Bananas, raw, sliced (150)	75
	Lunch	20013	Bulgur, cooked (91)	91
		4053	Oil, olive, salad or cooking	6.75
		11283	Onions, cooked, boiled, drained, without salt (210)	52.5
		11529	Tomatoes, red, ripe, raw, year round average, cherry tomatoes (149)	37.25
		11478	Squash, summer, zucchini, includes skin, cooked, boiled, drained, without salt, sliced (180)	45
		11457	Spinach,raw (60)	15
		11179	Corn, sweet, yellow, frozen, kernels cut off cob, boiled, drained, without salt (165)	82.5
		9152	Lemon juice, raw	5.9
		9266	Pineapple, raw, all varieties, chunks (165)	82.5
	Dinner	20125	Pasta, whole-wheat, cooked , penne (48.5)	48.5
		11547	Tomato products, canned, puree, without salt added (250)	62.5
		11365	Potatoes, boiled, cooked in skin, flesh, without salt chopped	156
		11125	Carrots, cooked, boiled, drained, without salt (156)	78
		11305	Peas, green, cooked, boiled, drained, without salt (160)	40
		11092	Broccoli, frozen,chopped, unprepared (156)	39
		1168	Cheese, low fat, cheddar or colby, shredded (37.7)	18.85
		9302	Raspberries, raw (120)	60
		1235	Yogurt, frozen, flavors not chocolate, nonfat milk, with low-calorie sweetener (136)	136
	Snacks	8037	Cereals ready-to-eat, granola, homemade (122) Made with oats, raisins, sunflower seeds, almonds, wheat germ, honey, coconut and canola oil.	61
		1256	Yogurt, Greek, plain, nonfat (170)	85
		9050	Blueberries, raw (148)	74
		16398	Peanut butter, smooth style, without salt (16)	16
		18216	Crackers, crispbread, rye, 2 crispbread (20)	20
		16158	Hummus, commercial (2 tbls) (30)	15

Energy intakes and outliers

We report below the results of our analysis when using different cut-offs for energy. In fact, we used a pragmatic range 200 - 1200 kcal in order to exclude extreme values. However, one could also exclude outliers based on some data-driven approach. For example, based on Tukey's definition, outliers can be defined as values outside of the interval:

$$[Q_1 - k(Q_3 - Q_1), Q_3 + k(Q_3 - Q_1)]$$

where Q_1 and Q_3 are respectively the first and third quartiles. A common choice for k is 1.5.

Table 5 below shows the distributions of energy intakes after excluding outliers. Using $k = 1.5$, non-outliers fall in the range $0 - 1262$ kcal.

Table 5: Quantiles of energy intakes of NHANES meals (kcal), after excluding outliers.

**Quartile**	**0%**	**25%**	**50%**	**75%**	**100%**
kcal	0	120	292	518	1262

Below, we report the results of the linear regression model after excluding outliers (with $k = 1.5$), therefore taking only meals with energy content in below 1262 kcal. The correlation between predicted and actual HEI on test data was 0.6.

Table 6: Linear regression model for HEI2010 as a function of MBI

	**Coefficient estimate**	**Standard error**	**p-value**
Breakfast	0.22	0.02	$<$ 2e-16
Lunch	0.19	0.02	1.51e-14
Snack	0.28	0.02	$<$ 2e-16
Dinner	0.15	0.02	1.54e-09
Age	0.13	0.01	$<$ 2e-16
Gender (female)	-0.15	0.69	0.82
Energy	-0.001	0.001	0.08

The optimal Ridge regularization parameter was selected by cross-validation and was estimated to be 0.03.

Table7 below replicates table 7 in the analysis, using meals in the range $0 - 1262$ kcal.

Table 7: Association between lunch scores and density of food groups and nutrients. All units are expressed per 2000 kcal. Different letters indicate statistically different distributions (pairwise Wilcoxon test, 95% confidence level). Recommendations based on the US dietary guidelines, when applicable.

**Food groups**	**Tertile of meal score**			**Unit**	**Recommendation.**
	[0, 43]	(43, 55]	(55, 100]		
Dairy	2.3^a	1.9^b	1.7^b	cup-eq/2000 kcal	3 cup-eq
Fruits	1^a	1.4^b	2^c	cup-eq/2000 kcal	2 cup-eq
Citrus, melons, berries	0.1^a	0.3^b	0.5^c	cup-eq/2000 kcal	
Grains	5.9^a	8.1^b	10.2^c	oz-eq/2000 kcal	6 oz-eq
Whole Grains	0.4^a	0.9^b	1.8^c	oz-eq/2000 kcal	3 oz-eq
Nuts and seeds	0.2^a	0.4^b	0.7^c	oz-eq/2000 kcal	
Solid fats	37^a	25^b	17^c	g/2000 kcal	
**Vitamins**					
Folate	323^a	468^b	525^c	μg/2000 kcal	400 μg
Vit A	628^a	764^b	748^c	μg/2000 kcal	900 μg
Vit C	87^a	123^b	146^c	mg/2000 kcal	90 mg
Vit E	7.2^a	8^a	8.7^b	μg /2000 kcal	15 μg
Vit K	168^a	202^b	184^c	μg/2000 kcal	120 μg
**Minerals**					
Copper	1^a	1.4^b	1.6^c	mg/2000 kcal	0.9 mg
Magnesium	224^a	298^b	348^c	mg/2000 kcal	420 mg
7.48 8.94 9.54 Zinc	7.5^a	8.9^b	9.5^c	mg/2000 kcal	11 mg
**Macronutrients**					
Energy	440^a	464^b	438^c	kcal	
Total sugar	157^a	123^b	107^c	g/2000 kcal	
Cholesterol	139^a	115^b	23^c	mg/2000 kcal	
